# Evidence of Purifying Selection and Co-Evolution at the Fold-Back Arm of the Novel Precursor MicroRNA159 Gene in *Phalaenopsis* Species (Orchidaceae)

**DOI:** 10.1371/journal.pone.0114493

**Published:** 2014-12-03

**Authors:** Chi-Chu Tsai, Yu-Chung Chiang, I-Szu Weng, Yu-Shium Lin, Chang-Hung Chou

**Affiliations:** 1 Kaohsiung District Agricultural Research and Extension Station, Pingtung, 908, Taiwan; 2 Department of Biological Science and Technology, National Pingtung University of Science and Technology, Pingtung, 912, Taiwan; 3 Department of Biological Sciences, National Sun Yat-sen University, Kaohsiung, 804, Taiwan; 4 Research Center for Biodiversity, China Medical University, Taichung, 404, Taiwan; National Cheng-Kung University, Taiwan

## Abstract

**Background:**

MicroRNAs (miRNAs) are small, endogenously transcribed, non-protein-coding RNAs that play important roles in regulation of gene expression in animals and plants. Here, selective constraints on the novel precursor microRNA159 (pre-miR159) gene were investigated in 42 *Phalaenopsis* species (Orchidaceae).

**Methods/Results:**

A novel precursor microRNA159 gene was isolated from 42 *Phalaenopsis* species using a new microRNA-PCR (miR-PCR) approach. Sequencing of pre-miR159 genes revealed differences from the canonical pre-miR159 gene in *Phalaenopsis* species and other plants. Results demonstrated that the 5′ and 3′ fold-back arms and the terminal loop of the novel pre-miR159 gene have undergone purifying selection and selective constraint for stabilizing the secondary hairpin structure. Two conserved motifs within the 5′ fold-back arm had the highest purifying selective pressure within the novel pre-miR159 gene. Evidence of sequence co-evolution between the 5′ and 3′ fold-back regions was observed.

**Conclusions:**

Functional selective pressure might arise from the constraint of forming a hairpin structure and demonstrate co-evolution of sequences between the 5′ and 3′ fold-back regions of the novel pre-miR159 gene in *Phalaenopsis* species.

## Background

MicroRNAs (miRNAs) are small, endogenously transcribed, non-protein-coding RNAs that regulate gene expression in animals and plants. Most mature miRNA are transcribed as independent transcriptional units and are approximately 20–24 nucleotides (nt) long. During miRNA processing in plants, primary miRNAs (pri-miRNAs) are transcribed by RNA polymerase II. After transcription, pri-miRNAs are processed into precursor miRNAs (pre-miRNAs) by an RNase III-like enzyme called DICER-LIKE 1 (DCL1) [1]–[4]. Processing by DCL1 releases approximately 22-base-pair (bp) imperfect RNA duplex intermediates (miRNA/miRNA* duplexes) [5]. Duplexes are exported to the cytoplasm where the RNA-induced silencing complex (RISC) produces one mature miRNA from the miRNA/miRNA* duplex. The strand selected to produce the mature miRNA within RISC is biased towards the duplex strand with the weakest hydrogen bond at its 5′ end. This weakly bonded strand is selectively incorporated into RISC [6]. Once mature, miRNAs down-regulate gene expression by mediating cleavage of mRNA and translational repression. To date, miRNAs have been found in a wide range of eukaryotes, including fruit flies, nematodes, zebrafish, chicken, mice, humans, *Arabidopsis*, maize, and rice [1].

Plant miRNAs recognize target mRNAs with near perfect base pairing. Therefore, computational sequence similarity searches can be used to identify potential targets [7]. In animals and plants, miRNAs are grouped into families where members differ by only a few nucleotides. Although family members are encoded at different loci, they are predicted to regulate similar or identical mRNAs [8]. Plant miRNAs can be encoded by the 5′ or 3′ fold-back arm of the hairpin. However, when miRNAs are encoded by multiple miRNA genes, miRNAs are always encoded by the same fold-back arm of the hairpin [9]. Early data suggested that plant miRNAs were conserved between monocots and dicots [10]. Indeed, twenty highly conserved miRNA families have been identified in three sequenced plant genomes: *Arabidopsis thaliana*, *Oryza sativa*, and *Populus trichocarpa*. However, deep-sequencing analyses revealed that most miRNAs are not conserved. In addition, although conserved miRNAs are often highly expressed [11], [12], copy numbers of miRNA genes are variable. Some families, including miR156 and miR159, contain numerous members in *A. thaliana*, *O. sativa*, and *P. trichocarpa*, whereas other families, such as miR162 and miR166, contain only a few genes [4].

The miR159 family is found in plants of the Embryophyta, Tracheophyta, Spermatophyta, Angiosperms, and Eudicots, indicating ancient origins [13]. According to miRBase, the miR159 family is encoded by multiple genes in *A. thaliana* [14], *O. sativa* [10], and *Glycine max* [15], [16] and by a single gene in *Arachis hypogaea* [17], *Festuca arundinacea* [18], and *Citrus sinensis* [19]. Single-stranded miR159 is 21 nt long and is always derived from the 3′ fold-back arm of the pre-miR159 family in *Arabidopsis*, *Oryza*, and *Populus* [4]. In *Arabidopsis*, miR159 mediates cleavage of MYB transcription factor mRNA in the germinating seed [20] and regulates flowering time and other developmental events [21], [22]. Although miR319 resembles miR159 [4], indicating both miRNA genes evolved from a common ancestor [23], they have distinct target mRNAs [24].

To understand the evolutionary pattern of the fold-back arm of miRNAs in plants, several different pre-miRNA gene sequences and structures have been surveyed [23], [25]–[27]. Comparing pre-miRNA gene sequences between closely related species should help determine molecular evolution patterns [25] and address the role of selective constraint [28]. In addition, both pre-miR159 and pre-miR319 are firstly processed from the loop of hairpin structure by DCL1. This separates them from other plant miRNAs, which are processed via first cutting at the base of hairpin structure [29]. Here, molecular evolution patterns and functional constraint of the pre-miR159 gene might help to determine the origins of the unique miRNA processing pattern of pre-miR159. A new analytical approach, microRNA-PCR (miR-PCR), was developed to examine the different regions of the pre-miR159 gene in 42 *Phalaenopsis* species (Orchidaceae), an ornamental flowering plant found distributed throughout tropical Asia and the Pacific Islands [30] for which molecular phylogenies were reconstructed [31], [32]. Analyses were designed to determine whether selective pressure has acted on the sequence or the hairpin structure of the pre-miR159 gene during evolution.

## Results and Discussion

### Isolation of the novel pre-miR159 gene from *Phalaenopsis* species

A single band was amplified from each *Phalaenopsis* species using miR-PCR. Five clones were randomly selected for clone-based sequencing. Sequences suggested the pre-miR159 gene might be encoded at a single locus in *Phalaenopsis* species with the exception of *P. sumatrana*, *P. lindenii*, and *P. gibbosa*, which had distinct sequences in the hairpin region. To further validate sequences of the miRNA/miRNA* duplexes for each *Phalaenopsis* species, five clones were randomly selected for amplification by inverse PCR (iPCR) and clone-based sequencing. Results showed a single miRNA/miRNA* duplex for each species. To validate these results, thirty clones were randomly selected from *P. amabilis* and products from miR-PCR and iPCR were sequenced. Results supported the claim that the PCR product amplified by miR-PCR was homogeneous.

**Figure 1:**
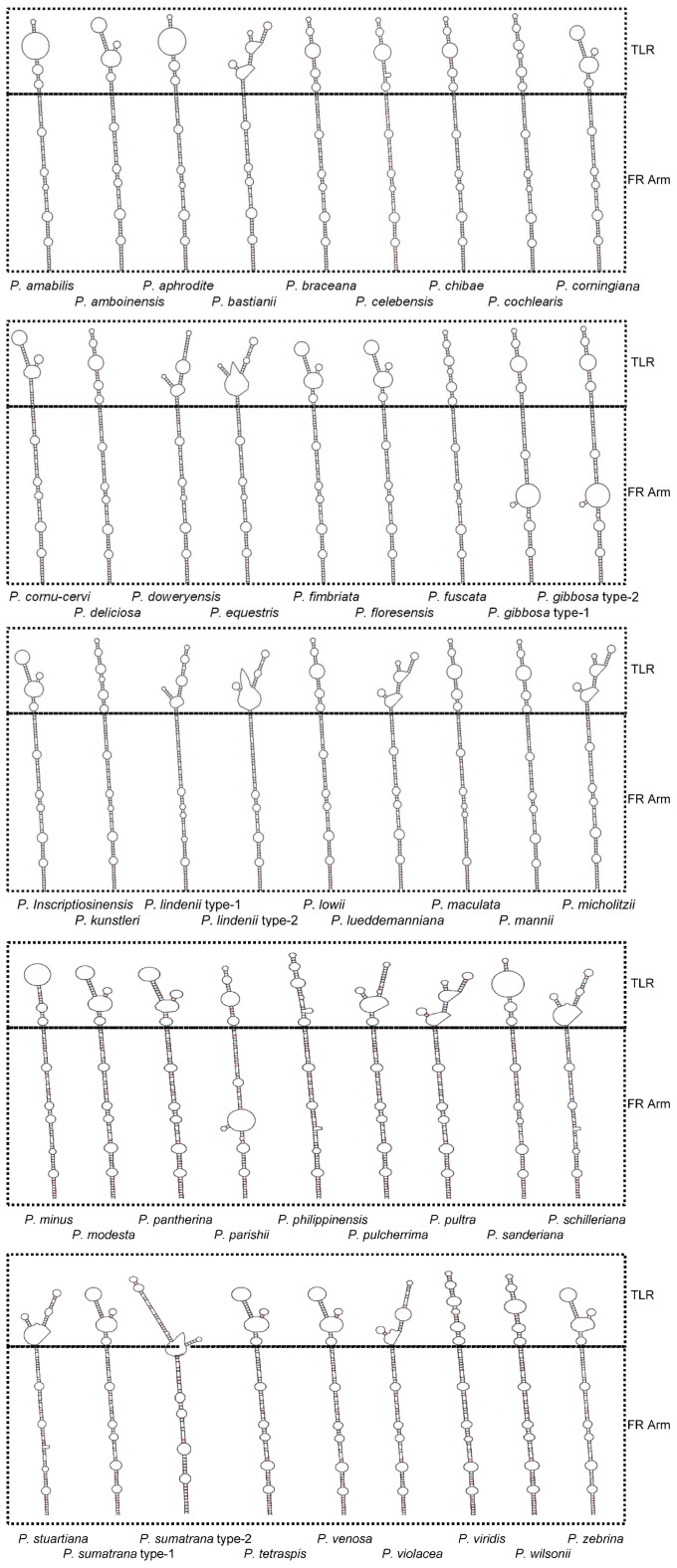
The hairpin secondary structure of the novel pre-miR159 from the 42 *Phalaenopsis* species. FR Arm means the fold-back arm and TLR means the terminal loop region.

The sequence of the pre-miR159 gene from *Phalaenopsis* species differed from other plants, and the second miR159, miR159.2, present in other plants, was not found [33], [34]. To characterize the differences, the *Phalaenopsis amabilis* canonical pre-miR159 gene was isolated by genome walking; miR159 primers were used for targeting. Secondary structures of the canonical and novel pre-miR159 genes from *P. amabilis* are provided in Figure S1. Higher base pairing in the secondary structure (65.8%) of the canonical pre-miR159 gene was observed compared to the novel pre-miR159 gene (53.9%). This observation may explain why the canonical pre-miR159 gene was not amplified by miR-PCR [35]. Secondary structures of the novel pre-miR159 for all *Phalaenopsis* species were predicted in Figure 1, and a close-up view of the pre-miRNA secondary structure is illustrated in Figure S2.

### Nucleotide sequence diversity of the novel pre-miR159 gene from *Phalaenopsis* species

**Figure 2:**
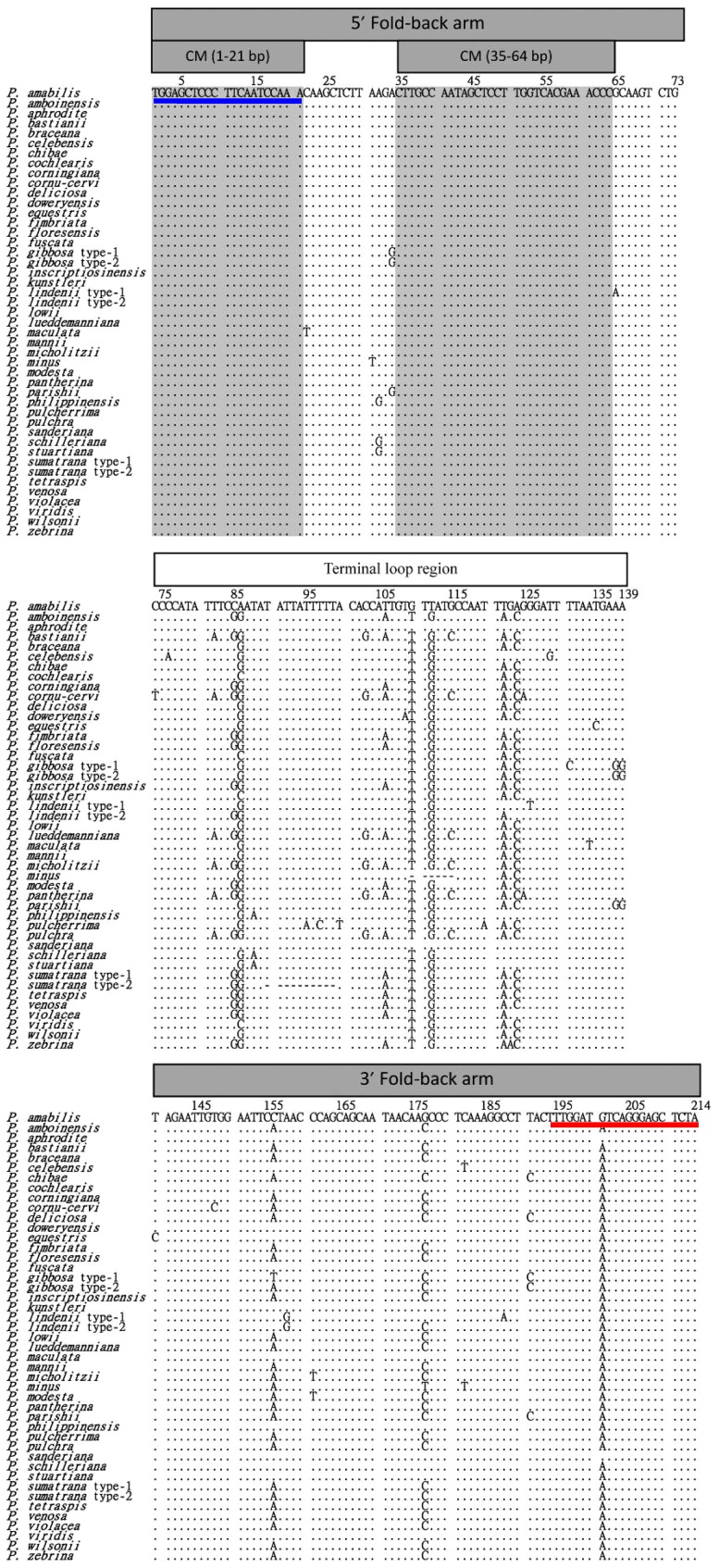
Nucleotide polymorphisms in the novel pre-miR159 gene among the 42 *Phalaenopsis* species. Nucleotides identical to the first line are indicated by a dot. Only base substitutions are indicated; deletion polymorphisms are indicated by dashes. The numbers at the top of the sequences represent the nucleotide positions. Positions 1 to 73, 74 to 139, and 140 to 214 represent the 5′ fold-back arm, the terminal loop region, and the 3′ fold-back arm, respectively. The gray regions are the longer highly conserved motifs among the 42 *Phalaenopsis* species. Blue and red lines, represent the predicted miRNA* and miRNA.

Nucleotide sequences of the novel pre-miR159 genes from 42 *Phalaenopsis* species were aligned. Of 214 sequenced bases, 42 sites were variable, including 19 single mutations (Figure 2, Table 1). Based on sequence alignments (Figure 2) and secondary structure predictions (Figure 1), positions 1 to 73 and 140 to 214 were critical for stabilizing the hairpin structure. Therefore, segments 1 to 73, 74 to 139, and 140 to 214 represented the 5′ fold-back arm, the terminal loop region, and the 3′ fold-back arm, respectively (Figure 2, Table 1).

**Table 1:** Pattern of nucleotide substitutions, including number of variable sites, nucleotide diversity (θ), and number of single mutations in the novel pre-miR159 gene (including 5′ fold-back arm, terminal loop region, and 3′ fold-back arm) and the internal transcribed spacer 1 (ITS1) of nuclear ribosomal DNA among 42 *Phalaenopsis* species.

DNA region	Positions	No. of sites	No. of variable sites	Nucleotide diversity (θ)	No. of single mutations
pre-miR159	1..214	214	42	0.044	19
5′ fold-back arm	1..73	73	5	0.016	3
Terminal loop region	74..139	66	27	0.093	13
3′ fold-back arm	140..214	75	10	0.031	3
ITS1 of ribosomal DNA	1..266	266	87	0.110	33

**Figure 3:**
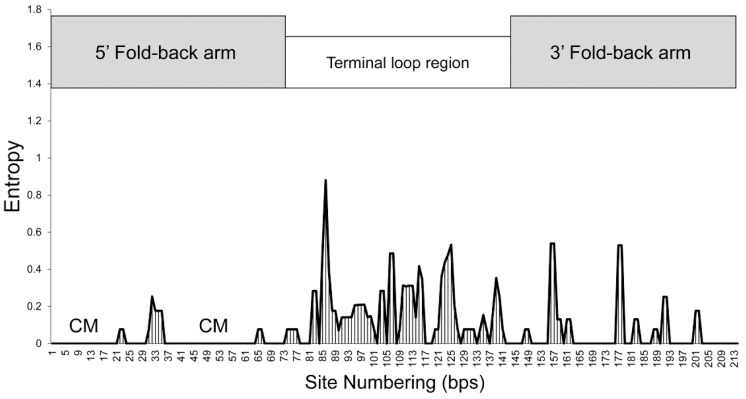
Substitution rate at each site within the novel pre-miR159 gene of 42 *Phalaenopsis* species. For each alignment of 42 sequences, the nucleotide substitution rate at each site was estimated by calculating entropy using the DAMBE software. A schematic of the gene is illustrated above the graph; fold-back arms are indicated as gray boxes and the terminal loop region is indicated as an open box. Two spikes in apparent diversity can be observed over the terminal loop region. Two conserved motifs (CM) are present in the 5′ fold-back arm.

The nucleotide diversity was θ = 0.044 for the entire novel pre-miR159 gene. The nucleotide diversity in the 5′ fold-back arm, the terminal loop, and the 3′ fold-back arm were θ = 0.016, 0.093, and 0.031, respectively. The nucleotide diversity in the terminal loop region was higher compared to the 5′ and 3′ fold-back arms, but it was close to that of the internal transcribed spacer 1 (ITS1) of nuclear ribosomal DNA (nrDNA) (Table 1). Substitution rates along the novel pre-miR159 gene revealed major differences between the three regions (Figure 3). High, variable substitution rates produced diversity in the terminal loop region, whereas two conserved motifs, 1 to 21 and 35 to 64, were present in the 5′ fold-back arm. Compared to the 3′ fold-back arm or the terminal loop region, the 5′ fold-back arm was more conserved. In the terminal loop region, one-third of the nucleotides were variable (27 variable sites/66 sites). These results suggest variability in the novel pre-miR159 gene was non-random.

Pair-wise nucleotide differences between species ranged from 0 to 0.0758 (average: 0.0276), 0 to 0.0282 (average: 0.0052), 0 to 0.0701 (average: 0.0231), 0 to 0.1891 (average: 0.0627), and 0 to 0.3688 (average: 0.0965) for the entire novel pre-miR159 gene, the 5′ fold-back arm, the 3′ fold-back arm, the terminal loop region, and the ITS1 of nrDNA, respectively. Nucleotide differences in the novel pre-miR159 genes were compared to the ITS1 of nrDNA, a neutral locus. Matched-pairs t-tests revealed significantly lower values for pair-wise nucleotide differences between species across the entire gene and within the three regions of the novel pre-miR159 genes (*p*<0.001). The maximum-likelihood relative rate test rejected the null hypothesis of rate constancy for 118 of 990 comparisons between *Paraphalaenopsis* outgroups and novel pre-miR159 genes (Table S1). Zero comparisons and few comparisons rejected the null hypothesis of rate constancy in the 5′ fold-back arm and 3′ fold-back arm (0 of 990 and 20 of 990 comparisons), respectively, but 140 of 990 comparisons rejected the null hypothesis of rate constancy in the terminal loop region (Table S2–S4). In instances where the null hypothesis was rejected, the terminal loop regions exhibited higher rates of nucleotide substitutions compared to the 5′ and 3′ fold-back arms. According to the definition of selective constraint described by Kimura and Takahata, these results indicate stronger purifying selection acted during evolution of the 5′ fold-back arm compared to the 3′ fold-back arm or the terminal loop region [36]. Five, 27, and 10 variable sites were detected in the 5′ fold-back arm, terminal loop region, and 3′ fold-back arm, respectively (Table 1). Considering that evolutionary divergence of functional DNA is reduced by purifying selection [36], DNA regions that have undergone purifying selection should have fewer segregating sites compared with a linked neutral region [37]–[39]. These results demonstrate that the 5′ and 3′ fold-back arms have undergone purifying selection during evolution.

### Functional constraints of the novel pre-miR159 gene fold-back arms in *Phalaenopsis* species

According to sequence alignments of the novel pre-miR159 genes of 42 *Phalaenopsis* species (Figure 2), 15 segregating sites exist within the 5′ and 3′ fold-back arms (Table 2). Comparing nucleotide sequence substitutions (Figure 2) and secondary hairpin structures (Figure 1), five newly formed base pairings which can increase the stability of the hairpin structure, and five synonymous base-pair substitutions (A-U→G-U, G-U→A-U, G-C→G-U, and G-U→G-C) were found [40]. Other substitutions located in internal loops, that did not affect the hairpin structure, were also discovered (Table 2). These results indicate that the substitutions found within the 5′ and 3′ fold-back arms do not destroy secondary structure. These results suggest that the novel pre-miR159 gene was randomly mutated during evolution, but that only substitution events that did not destroy secondary structure were retained and inherited. These results also indicated that functional constraints were present during evolutionary processing of the fold-back regions and demonstrate co-evolution of the 5′ and 3′ fold-back regions.

**Table 2:** Nucleotide substitutions within the fold-back arms of the novel pre-miR159 genes and the corresponding pairing in the secondary structure among 42 *Phalaenopsis* species.

Taxa	Sequence substitution in the novel pre-miR159 gene fold-back regions		
	Position^a^	Substitution type	Nucleotide of corresponding pair in the secondary structure
*P. maculata*	22	C→U*	A
*P. minus*	31	A→U*	A
*P. schilleriana, P. stuartiana, P. philippinensis*	32	A→G**	U
*P. gibbosa*-type-1, *P. gibbosa*-type-2, *P. parishii*	34	A→G***	Internal loop
*P. lindenii*-type-1	65	G→A**	U
*P. equestris*	140	U→C**	G
*P. cornu-cervi*	148	U→C**	G
14 species (*P. gibbosa*-type-1)	156	A→C*** (U***)	Internal loop
*P. lindenii*-type-1, *P. lindenii*-type-2	158	A→G*	C
*P. modesta*, *P. micholitzii*	161	C→U**	G
14 species (*P. minus*)	177	C→G*(U***)	C (Internal loop)
*P. celebensis*, *P. minus*	182	C→U*	A
*P. lindenii*-type-1	188	C→A***	C
*P. chibae, P. deliciosa, P. parishii, P. gibbosa*-type-1, *P. gibbosa*-type-2	191	U→C***	Internal loop
*P. amabilis, P. aphrodite, P. sanderiana*	201	A→G***	Internal loop

a Nucleotide position according to Figure 1. Positions 1–73, 74–139, and 140–214 represent the 5′ fold-back arm, the terminal loop region, and the 3′ fold-back arm, respectively.

*Represents increasing stability (new base pairing) of the fold-back structure; ** Represents a minor change in fold-back stability (increase or decrease) without destroying the nucleotide pairing (i.e., A-U→G-U, G-C→G-U, G-U→G-C, or G-U→A-U); *** Represents no change in the fold-back stability or substitutions located in the internal loops

Secondary structure of the terminal loop in the novel pre-miR159 gene was variable among 42 *Phalaenopsis* species examined, although the stem structure of fold-back arms was conserved (Figure 1). No insertions/deletions (indels) were observed within the 5′ or 3′ fold-back arms of the novel pre-miR159 gene in each of 42 *Phalaenopsis* species examined (Figures 1 and 2). In contrast, indels were found in the terminal loop region in two taxa, *P. minus* and *P. sumatrana* (Figure 2). The novel pre-miR159 gene in *P. minus* contained a 6 nt deletion in the terminal loop, the fold-back structure resembled the novel pre-miR159 gene from other *Phalaenopsis* species (Figures 1 and 2; Figure S3). However, the conserved stem structure of fold-back arms was destroyed in *P. sumatrana* by a 10 nt deletion (positions 90 to 99 in the alignment sequence) (Figures 1 and 2; Figure S3). It is unclear whether the novel pre-miR159 gene in *P. sumatrana* can produce a mature miR159. However, several studies have shown that efficiency of miRNA production is reduced when the fold-back structure is destroyed [25], [41], [42]. These results indicate that the buffering capacity of indels in the terminal loop is higher compared to the arms. This may be because the structure of both arms is necessary for pre-miRNA processing [1]–[4].

To determine whether the secondary structure or the sequence of the novel pre-miR159 gene has any highly conserved regions, all predicted secondary structures and sequence alignments of the novel pre-miR159 genes from *Phalaenopsis* were compared (Figures 1 and 2). Two conserved internal loops, one within the miR159/miR159* duplex and one near the 5′-end of miR159, were observed. Two highly conserved motifs within the 5′ fold-back arm were also found. *Arabidopsis* DCL1, a miRNA processing protein, contains two double-strand RNA binding domains (dsRBDs); one plays a major role in pri-miRNA binding [43]. Selective constraint of the novel pre-miR159 5′ fold-back arm may be involved in DCL1 targeting, suggesting that conserved structures and sequences in the novel pre-miR159 gene may play important roles in the unique processing that has been described [29].

The first conserved motif is the complementary sequence (21 nt) of miR159. The second is located within the 5′ fold-back arm at positions 35 to 64 (30 nt) (Figures 1 and 2). This result is consistent with the canonical pre-miR159 and pre-miR319 found in other plants [44]. The second conserved motif, found within the 5′ fold-back arm, is only found in the pre-miR159 and pre-miR319 families [44]. Also revealed by sequence alignments, the nucleotide sequence of the second miRNA derived from the canonical pre-miR159 gene is not conserved among plants. The second miRNA derived from pre-miR159 is also processed differentially among plants. In *Phaseolus vulgaris,* the second miRNA is processed in response to stress [27], [34]. It has also been observed from the canonical pre-miR159 in *Phalaenopsis aphrodite* subspecies *formosana* by deep sequencing [45]. Because miRNA* is selectively constrained for miRNA biogenesis [3], selective constraint for the second conserved motif of the novel pre-miR159 gene may come from processing of the second miR159.

### Evolution of the novel miR159 gene in *Phalaenopsis* species

**Figure 4:**
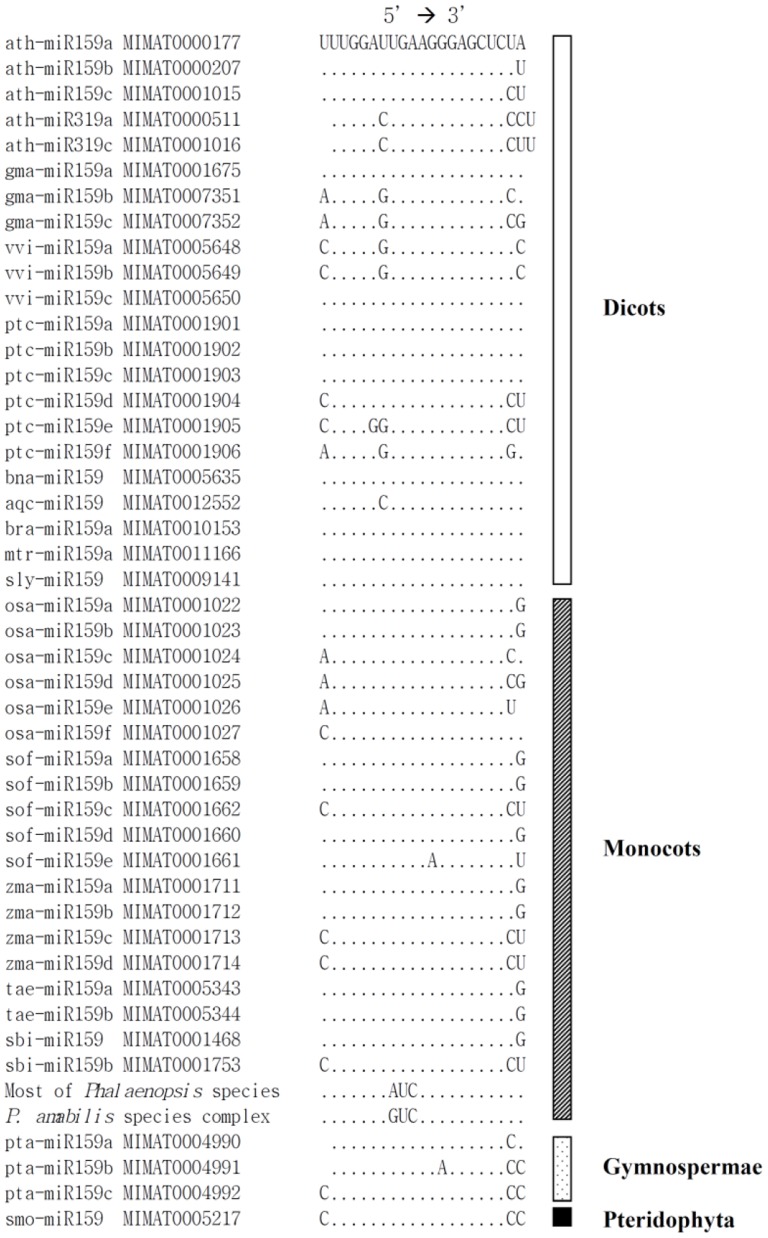
A comparison of the predicted miR159 derived from the novel pre-miR159 genes of the *Phalaenopsis* species and members of the miR159 family from diverse plants. Positions 8–10 from the 5′ end of the miR159 derived from the *Phalaenopsis* species indicate three distinct similarities with other members of the miR159 family. Two types of miR159 can be observed across the *Phalaenopsis* species. Position 8 is adenosine (A) in most of the *Phalaenopsis* species, whereas in other species (the *Phalaenopsis amabilis* species complex, which includes *P. amabilis*, *P. aphrodite*, and *P. sanderiana*) it is guanosine (G).

In *Arabidopsis*, *Oryza*, and *Populus,* the miR159 family is derived from the 3′ fold-back arm of the pre-miRNA [4]. The predicted miRNA159 derived from the novel pre-miR159 gene is 5′-UUUGGAUAUCAGGGAGCUCUA-3′; however, three *Phalaenopsis* species (*P. amabilis*, *P. aphrodite*, and *P. sanderiana*) have a substitution at position 8 from the 5′ end (i.e., 5′-UUUGGAUGUCAGGGAGCUCUA-3′, the substitution is underlined). The morphological characteristics of these *Phalaenopsis* species are distinct from other members of the section *Phalaenopsis*, including *P. philippinensis*, *P. schilleriana*, and *P. stuartiana*, which have marbling on the upper surface of their leaves and bear anthocyanins in the leaves [30]. The two predicted miR159 derived from the novel pre-miR159 genes in *Phalaenopsis* was aligned with other members of the miR159 family from miRBase (Figure 4). According to alignments, the length of miR159 was 21 or 20 nt, and the 5′ and 3′ ends were more variable. In addition, alignments revealed no correlation between sequence divergence and phylogenetic relationships, suggesting that miR159 family members have experienced high levels of selective constraint during evolution.

The predicted miR159 from three species, *P. sumatrana*, *P. lindenii*, and *P. gibbosa*, may be processed from two copies of the novel pre-miR159 gene. This was indicated by variations (substitutions or deletions) observed in the terminal loop region of the novel pre-miR159 gene in these species. These three non-conserved paralogs of the novel pre-miRNAs may be considered young copies [13], which would be consistent with the model that miRNAs are created and destroyed continuously during evolution [11], [46]. This result also indicates that a duplication event occurred in these three species.

The canonical pre-miR159 gene from *P. amabilis* was further isolated by genome walking (Figure S1). The miR159 (5′-UUUGGAUUGAAGGGAGCUCUA-3′) derived from the canonical pre-miR159 gene of *P. amabilis* is typical [9] (Figure 4) and abundantly expressed in *P. aphrodite* subspecies *formosana* [45]. The novel pre-miR159 genes isolated from all *Phalaenopsis* species can form hairpin structures and are subject to selective constraint for stabilizing the fold-back structure. Therefore, the novel pre-miR159 gene may elicit biological function by generating miRNAs that down-regulate target mRNA.

Although miRNA and its target mRNA require near-perfect base pairing in plants [7], three substitutions between novel miR159 and canonical miR159 were discovered. These changes, located in the seed region (defined as the second to the seventh nucleotides in the mature miRNA) which are critical for target recognition [47]. Indeed, both canonical miR159 and its target site can be found on the *MYB* transcript in *Phalaenopsis* species [45], [48]. These data indicate new miRNA genes may evolve by point mutation and selection against inadequate miRNA/mRNA pairing [49]. Therefore, the predicted novel miR159 might target and down-regulate other unknown mRNAs. Two other possibilities may explain the novel miR159 sequence observed in *Phalaenopsis* species. First, novel miR159 cannot be accurately processed from the novel pre-miR159 gene due to tissue-specific control of miRNA processing [50], [51]. This is supported by the absence of novel miR159 in leaf tissues of *P. aphrodite* subspecies *formosana* [45]. Second, RNA editing of pre-miRNA/miRNA has been observed in several studies [52]–[54]. Therefore, novel pre-miR159 might undergo RNA editing to generate canonical miR159 in *Phalaenopsis* species.

### Conclusions

A novel pre-miR159 gene was isolated from *Phalaenopsis* species. The nucleotide sequence of the novel pre-miR159 gene differed from the canonical pre-miR159 gene in *Phalaenopsis* species and other plants. Regions of the novel pre-miR159 gene were associated with distinct purifying selective pressures. The 5′ fold-back arm displayed evidence of strong purifying selection during evolution, and the 5′ and 3′ fold-back arms were subject to selective constraints. Selective constraints were also indicated for the stem of the hairpin structure in the novel pre-miR159 gene, and evidence of co-evolution of the 5′ and 3′ fold-back regions was uncovered. Strong purifying selection of the 5′ fold-back arm implied that motifs in the region may be critical for miR159 processing and biogenesis. Moreover, it appears that the novel pre-miR159 gene has undergone duplication events.

## Methods

### Plant materials

Forty-two species were selected from the subgenera and sections of the genus *Phalaenopsis*. Leaf materials were collected from living plants cultivated in the Kaohsiung District Agricultural Research and Extension Station (KDARES) in Taiwan. Voucher specimens were deposited at the herbarium of the National Museum of Natural Science, Taiwan (TNM). Details of the materials, their distributions, and systematic classifications are listed in Table 3.

**Table 3:** Names of the specimens, geographical distributions, and sources for the plant materials in the study.

Taxa and systematic classification a	Geographical distribution	Voucher b
Genus *Phalaenopsis*		
**Subgenus ** ***Proboscidioides*** ** (Rolfe) E. A. Christ.**		
*Phalaenopsis lowii* Rchb.f. b	Myanmar and adjacent western Thailand	C. C. Tsai 1714
**Subgenus ** ***Aphyllae*** ** (Sweet) E. A. Christ.**		
*Phalaenopsis wilsonii* Rolfe	China (Sichuan, Yunnan, and eastern Tibet)	C. C. Tsai 1645
*Phalaenopsis minus* (Seidenf.) E. A. Christ.	Endemic to Thailand	C. C. Tsai 1791
*Phalaenopsis braceana* (J. D. Hook.) E. A. Christ.	Bhutan and China	C. C. Tsai 1747
**Subgenus ** ***Parishianae*** ** (Sweet) E. A. Christ**.		
*Phalaenopsis gibbosa* Sweet	Vietnam and Laos	C. C. Tsai 1783
*Phalaenopsis parishii* Rchb.f.	Eastern Himalayas, India, Myanmar, and Thailand	C. C. Tsai 1316
**Subgenus ** ***Polychilos*** ** (Breda) E. A. Christ.**		
**Section ** ***Polychilos*** ** (Breda) Rchb.f.**		
*Phalaenopsis mannii* Rchb.f.	Northeast India, Nepal, and China to Vietnam	C. C. Tsai 1796
*Phalaenopsis cornu-cervi* (Breda) Bl. & Rchb.f.	Northeast India and the Nicobar Islands to Java and Borneo	C. C. Tsai 1562
*Phalaenopsis pantherina* Rchb.f.	Endemic to Borneo	C. C. Tsai 1395
**Section ** ***Fuscatae*** ** Sweet**		
*Phalaenopsis cochlearis* Holtt.	Malaysia (Malay Peninsula) and Indonesia (Sarawak)	C. C. Tsai 1722
*Phalaenopsis viridis* J. J. Sm.	Endemic to Indonesia (Sumatra)	C. C. Tsai 1141
*Phalaenopsis fuscata* Rchb.f.	Malaysia (Malay Peninsula), Borneo (West Koetai)	C. C. Tsai 1733
*Phalaenopsis kunstleri* J. D. Hook.	Myanmar and Malay Peninsula	C. C. Tsai 1139
**Section ** ***Amboinenses*** ** Sweet**		
*Phalaenopsis pulchra* (Rchb.f.) Sweet	Endemic to the Philippines (Luzon and Leyte)	C. C. Tsai 1281
*Phalaenopsis violacea* Witte	Indonesia (Sumatra) and Malaysia (Malay Peninsula)	C. C. Tsai 1598
*Phalaenopsis micholitzii* Rolfe	Philippines (Mindanao)	C. C. Tsai 1674
*Phalaenopsis fimbriata* J. J. Sm.	Indonesia (Java, Sarawak, and Sumatra)	C. C. Tsai 1732
*Phalaenopsis floresensis* Fowlie	Endemic to the island of Flores	C. C. Tsai 1610
*Phalaenopsis doweryensis* Garay & E. A. Christ.	East Malaysia (Sabah), without a precise locality	no voucher
*Phalaenopsis modesta* J. J. Sm.	Endemic to the island of Borneo in East Malaysia (Sabah) and Indonesia (Kalimantan)	C. C. Tsai 1756
*Phalaenopsis maculata* Rchb.f.	Malaysia (Pahang), East Malaysia (Sabah and Sarawak), and Indonesia (Kalimantan Timur)	C. C. Tsai 1774
*Phalaenopsis amboinensis* J. J. Sm.	Indonesia (Molucca Archipelago and Sulawesi)	C. C. Tsai 1605
*Phalaenopsis lueddemanniana* Rchb.f.	Endemic to the Philippines	C. C. Tsai 1162
*Phalaenopsis bastianii* Gruss & Rollke	Endemic to the Philippines	C. C. Tsai 1412
*Phalaenopsis venosa* Shim & Fowlie	Endemic to Indonesia (Sulawesi)	C. C. Tsai 1014
**Section ** ***Zebrinae*** ** Pfitz.**		
*Phalaenopsis inscriptiosinensis* Fowlie	Endemic to Indonesia (Sumatra)	no voucher
*Phalaenopsis tetraspis* Rchb.f.	India (Andaman and Nicobar Islands) and Indonesia (Sumatra)	C. C. Tsai 1693
*Phalaenopsis corningiana* Rchb.f.	Borneo (Sarawak and elsewhere on the island)	C. C. Tsai 1776
*Phalaenopsis sumatrana* Korth. & Rchb.f.	Widespread from Myanmar, Thailand, and Vietnam, to Indonesia (Java and Sumatra), Malaysia (Perak and Johore), East Malaysia (Sabah), and the Philippines (Palauan)	C. C. Tsai 1650
*Phalaenopsis zebrina* Witte		no voucher
**Subgenus ** ***Phalaenopsis***		
**Section ** ***Phalaenopsis*** ** Benth.**		
*Phalaenopsis philippinensis* Golamco ex Fowlie & Tang	Endemic to the Philippines	C. C. Tsai 1333
*Phalaenopsis amabilis* (L.) Blume	Widespread from Sumatra and Java to the southern Philippines, and east to New Guinea and Queensland, Australia	C. C. Tsai 193
*Phalaenopsis aphrodite* Rchb.f.	Northern Philippines and southeastern Taiwan	C. C. Tsai 1420
*Phalaenopsis sanderiana* Rchb.f.	Endemic to the Philippines	C. C. Tsai 1526
*Phalaenopsis schilleriana* Rchb.f.	Endemic to the Philippines	C. C. Tsai 1004
*Phalaenopsis stuartiana* Rchb.f.	Endemic to the island of Mindanao in the southern Philippines	C. C. Tsai 1419
**Section ** ***Deliciosae*** ** E. A. Christ.**		
*Phalaenopsis chibae* Yukawa	Endemic to Vietnam	C. C. Tsai 1792
*Phalaenopsis deliciosa* Rchb.f.	Widespread from Sri Lanka and India to the Philippines and Sulawesi	C. C. Tsai 1664
**Section ** ***Esmeralda*** ** Rchb.f.**		
*Phalaenopsis pulcherrima* (Lindl.) J. J. Sm.	Widespread from northeast India and southern China throughout Indochina to Malaysia (Malay Peninsula), Indonesia (Sumatra), and East Malaysia (Sabah)	C. C. Tsai 1020
**Section ** ***Stauroglottis*** ** (Schauer) Benth.**		
*Phalaenopsis equestris* (Schauer) Rchb.f.	Philippines and Taiwan	C. C. Tsai 1689
*Phalaenopsis celebensis* Sweet	Endemic to Indonesia (Sulawesi)	C. C. Tsai 1671
*Phalaenopsis lindenii* Loher	Endemic to the Philippines	C. C. Tsai 1574

a The systematic characterizations of *Phalaenopsis* are based on Christenson (2001).

b Their voucher specimens were deposited at the herbarium of the National Museum of Natural Science, Taiwan (TNM).

### Primer design and PCR amplification of the novel pre-miR159 gene

Genomic DNA was extracted from fresh *Phalaenopsis* leaves using the cetyltrimethylammonium bromide protocol [55]. To investigate sequence variation of the pre-miR159 gene between *Phalaenopsis* species, a new analytical approach, based on near-perfect base pairing and inverted repeats located at both ends of the pre-miRNA [7], were developed. Taking into account that the miRNA is located on the same fold-back arm (5′ or 3′) among diverse plants [4] and that the length of the mature miRNA is approximately 20-24 nt [1], [3], [4], a single primer was designed to amplify the pre-miRNA region. This approach was named microRNA-PCR (miR-PCR). Primers derived from the conserved miR159 region in *Arabidopsis* (ath-miR159a: 5′-UUUGGAUUGAAGGGAGCUCUA-3′) [9], [56] and *Oryza* (osa-miR159a: 5′-UUUGGAUUGAAGGGAGCUCUG-3′) [10] were designed to amplify the pre-miR159 region from *Phalaenopsis*. The conserved sequence of miR159a, 5′-UUUGGAUUGAAGGGAGCUCU-3′, is located on the 3′ fold-back arm. Consequently, the sequence of the single primer used for amplifying the novel pre-miR159 region of the *Phalaenopsis* species was 5′-AGAGCTCCCTTCAATCCAAA-3′.

PCR reactions (25 µl) contained 40 mM Tricine-KOH (pH 8.7), 15 mM KOAc, 3.5 mM Mg(OAc)_2_, 3.75 µg/ml BSA, 0.005% Tween 20, 0.005% Nonidet-P40, four dNTPs (0.2 mM each), primers (0.4 µM each), 1.25 U of Advantage 2 DNA polymerase (Clontech Laboratories, Inc., CA, USA), and 10 ng of genomic DNA. Cycling was performed in a thermocycler (Biometra, Germany) under the following conditions: 94°C for 5 minutes followed by 40 cycles of denaturation at 94°C for 40 seconds, annealing at 50°C for 35 seconds, and extension at 72°C for 50 seconds, with a final extension at 72°C for 7 minutes. PCR products were visualized on a 1% agarose gel. A product of the expected size was amplified from each of the samples. Amplified products were purified using Qiagen columns (Valencia, CA, USA), and purified PCR products were cloned into *p*GEM-T Easy Vectors (TaKaRa, Japan). Five independent clones were sequenced using the dideoxy chain-termination method and an ABI3730 automated sequencer with the BigDye Terminator Cycle Sequencing Ready Reaction Kit (PE Biosystems, CA, USA). Sequencing reactions were performed according to the manufacturer's recommendations.

### Inverse PCR

Inverse PCR (iPCR) was performed as described by Ochman *et al.* [57]. Universal primers for iPCR were designed within the hairpin structure, excluding the miRNA/miRNA* duplex: F, 5′-GTGGAATTCATAACCCAGTAGTA-3′ and R, 5′-GGGTTTCGTGACCAAGGAGCTA-3′. Nested primers for the second PCR were F, 5′-ATTCATAACCCAGCAGCAATAACA-3′, and R, 5′-CTATTGGCAAGTCTTAAGAGCTTG-3′. Genomic DNA from 42 *Phalaenopsis* species was digested with *Dra*I. DNA fragments were ligated to obtain intramolecular circularized DNA for iPCR amplification. PCR reactions (25 µl) contained 40 mM Tricine-KOH (pH 8.7), 15 mM KOAc, 3.5 mM Mg(OAc)_2_, 3.75 µg/ml BSA, 0.005% Tween 20, 0.005% Nonidet-P40, four dNTPs (0.2 mM each), primers (0.4 µM each), 1.25 U Advantage 2 DNA polymerase (Clontech Laboratories, Inc., CA, USA), and 10 ng genomic DNA. Cycling was performed in a thermocycler (Biometra) using the following conditions: 94°C for 5 minutes followed by 35 cycles of denaturation at 94°C for 40 seconds, annealing at 56°C for 40 seconds, and extension at 72°C for 2 minutes, with a final extension at 72°C for 7 minutes. PCR products were detected by agarose gel electrophoresis (1.0% w/v in TBE), stained with 0.5 µg/ml ethidium bromide, and photographed under UV light exposure. A product of the expected size was amplified from each of the samples, and amplified products were purified using Qiagen columns (Valencia, CA, USA). Purified PCR products were cloned into *p*GEM-T Easy Vectors (TaKaRa, Japan), and five independent clones were sequenced. Cloned DNA was sequenced following the dideoxy chain-termination method using an ABI3730 automated sequencer with the BigDye Terminator Cycle Sequencing Ready Reaction Kit (PE Biosystems, CA, USA). Sequencing reactions were performed according to the manufacturer's recommendations. All validated pre-miR159 sequences have been deposited into GenBank with the accession numbers GU166689-GU166733. To compare nucleotide substitutions between neutral and novel pre-miR159 gene sequences, sequences of the internal transcribed spacer 1 (ITS1) of nuclear ribosomal DNA (nrDNA) for these *Phalaenopsis* species published by Tsai *et al.* [31] were re-aligned. To estimate the relative rate test, two *Paraphalaenopsis* taxa (*Paraphalaenopsis laycockii* and *Paraphalaenopsis serpentilingua*) were included in the study as outgroups.

### Genome walking

Using miR159.2 as a primer targeting region, the canonical pre-miR159 gene was isolated using the Genome Walker Universal Kit (Clontech Laboratories, Inc., CA, USA). PCR was performed with Advantage 2 DNA polymerase (Clontech Laboratories, Inc., CA, USA). DNA fragments were purified with the QIAquick Gel Extraction Kit (Qiagen, Valencia, CA, USA). Recovered PCR products were ligated into T-vectors (Promega, Wisconsin, USA), and recombinants were transformed into *Escherichia coli* DH5α (RBC, Taipei, Taiwan). Plasmid DNA was purified using Qiagen spin mini prep kits. Plasmid DNA was sequenced with vector-specific primers (SP6 and T7) using the dideoxy chain-termination method, an ABI3730 automated sequencer, and the Ready Reaction Kit (PE Biosystems, CA, USA) for BigDye Terminator Cycle Sequencing. Each sample was sequenced three times. Reactions were performed according to the manufacturer's recommendations.

### Sequence alignment, secondary structure prediction, and nucleotide variability

Sequences of novel pre-miR159 genes were aligned using the Clustal W multiple alignment program in BioEdit [58]. The hairpin structure of the novel pre-miR159 was predicted using RNA folding software [59]. Alignment results coupled with the secondary structure of the novel pre-miR159 genes were used to guide the division of pre-miR159 into three regions: the 5′ fold-back arm, the terminal loop region, and the 3′ fold-back arm. To detect sequence polymorphisms for the different regions of pre-miR159 gene, the number of variable sites, nucleotide diversity (θ), and single mutations were estimated using DNASP version 4.10 [60].

### Substitution rate at each site (entropy) calculation

To evaluate variability and complexity of each nucleotide site, entropy for each nucleotide site was estimated using the Shannon entropy formula: 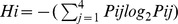 [61], where Hi corresponds to the entropy of each site I; j is equal to 1, 2, 3 and 4, corresponding to the A, C, G, and T nucleotides, respectively; and Pij is the proportion of nucleotide j in site i [62]. For entropy analyses, aligned sequences of the novel pre-miR159 genes were estimated using Data Analysis in Molecular Biology and Evolution (DAMBE) v. 5.2.76 [63].

### Determination of nucleotide substitutions per site (D_XY_) and the relative rate test

The number of nucleotide substitutions per site between species (D_XY_) was estimated using the six-parameter method [64]. Pair-wise nucleotide differences were calculated using DAMBE v.5.2.76 [63] for the entire novel pre-miR159 genes, the 5′ fold-back arm, the 3′ fold-back arm, the terminal loop region, and the ITS1 of nrDNA, respectively. To compare significance between the novel pre-miR159 gene and the ITS1 of nrDNA, statistical analyses were performed using matched-pairs t-tests for paired groups; *p* values of <0.05 were considered statistically significant. Maximum-likelihood relative rate tests were estimated using HyPhy version 2.10 [65]. Nucleotide substitution models were evaluated by hierarchical likelihood ratio tests implemented in Modeltest version 3.7 [66]. The Jukes and Cantor 1969 (JC69) model [67] was determined the best model by having the highest Bayesian Information Criterion (BIC) value. The relative rate test compares the number of nucleotide substitutions per site between two ingroup species by exploiting outgroups to classify those substitutions that can be unambiguously assigned to one of the ingroup taxa [68]. To test significance between different regions of pre-miR159 genes, statistical analyses using t-tests for paired groups were performed.

## Supporting Information

Figure S1 **The hairpin secondary structure of the novel pre-miR159 (A) and canonical pre-miR159 (B) in** ***Phalaenopsis amabilis*** **.**
(PDF)

Figure S2 **A close-up view of the secondary structure of the novel pre-miR159 from the 42** ***Phalaenopsis*** **species.**
(PDF)

Figure S3 **The hairpin secondary structure of the novel pre-miR159 from (A)** ***Phalaenopsis minus*** **; and (B)** ***P. sumatrana*** **-type 2 with a 10 nt deletion within the terminal loop region.** The blue line region represents the fold-back arm of secondary structure for all *Phalaenopsis* species. The red line region represents the mature miR159.
(PDF)

Table S1 **The maximum-likelihood relative rate test in the novel pre-miR159 gene of 42** ***Phalaenopsis*** **species.** Pairwise comparisons of Nucleotide substitution rate (above diagonal) and P-value (below diagonal) between species determined from the novel pre-miR159 gene.
(PDF)

Table S2 **The maximum-likelihood relative rate test in the 5' Fold-back arm of the novel pre-miR159 gene of 42** ***Phalaenopsis*** **species.** Pairwise comparisons of Nucleotide substitution rate (above diagonal) and P-value (below diagonal) between species deduced from the 5' Fold-back arm of the novel pre-miR159 gene.
(PDF)

Table S3 **The maximum-likelihood relative rate test in the terminal loop region of the novel pre-miR159 gene of 42** ***Phalaenopsis*** **species.** Pairwise comparisons of Nucleotide substitution rate (above diagonal) and P-value (below diagonal) between species deduced from the terminal loop region of novel the pre-miR159 gene.
(PDF)

Table S4 **The maximum-likelihood relative rate test in the 3' Fold-back arm of the novel pre-miR159 gene of 42** ***Phalaenopsis*** **species.** Pairwise comparisons of nucleotide substitution rate (above diagonal) and P-value (below diagonal) between species deduced from the 3' Fold-back arm of the novel pre-miR159 gene.
(PDF)
