# Supplementary figures and images for: Evidence of Purifying Selection and Co-Evolution at the Fold-Back Arm of the Novel Precursor MicroRNA159 Gene in Phalaenopsis Species (Orchidaceae)
Source: PLoS One. 2014 Dec 3;9(12):e114493. doi: 10.1371/journal.pone.0114493 (PMC4254996; doi:10.1371/journal.pone.0114493)

Fig. S1

(A)

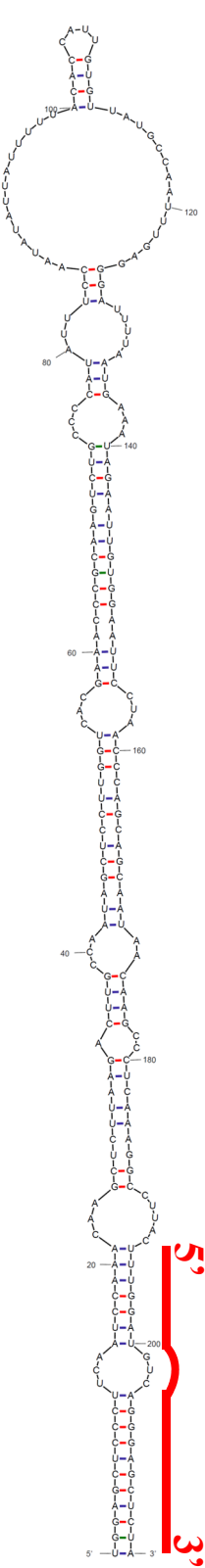

Novel type

(B)

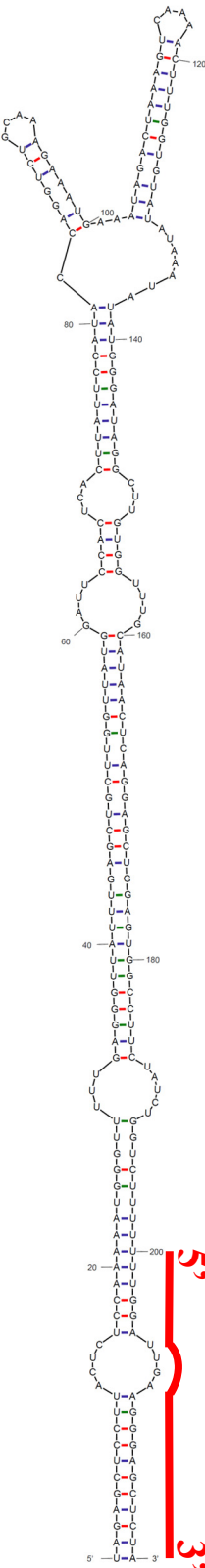

Canonical type

Supplement: Figure S1 — The hairpin secondary structure of the novel pre-miR159 (A) and canonical pre-miR159 (B) in Phalaenopsis amabilis . (PDF) [file pone.0114493.s001.pdf]

**Fig. S3**

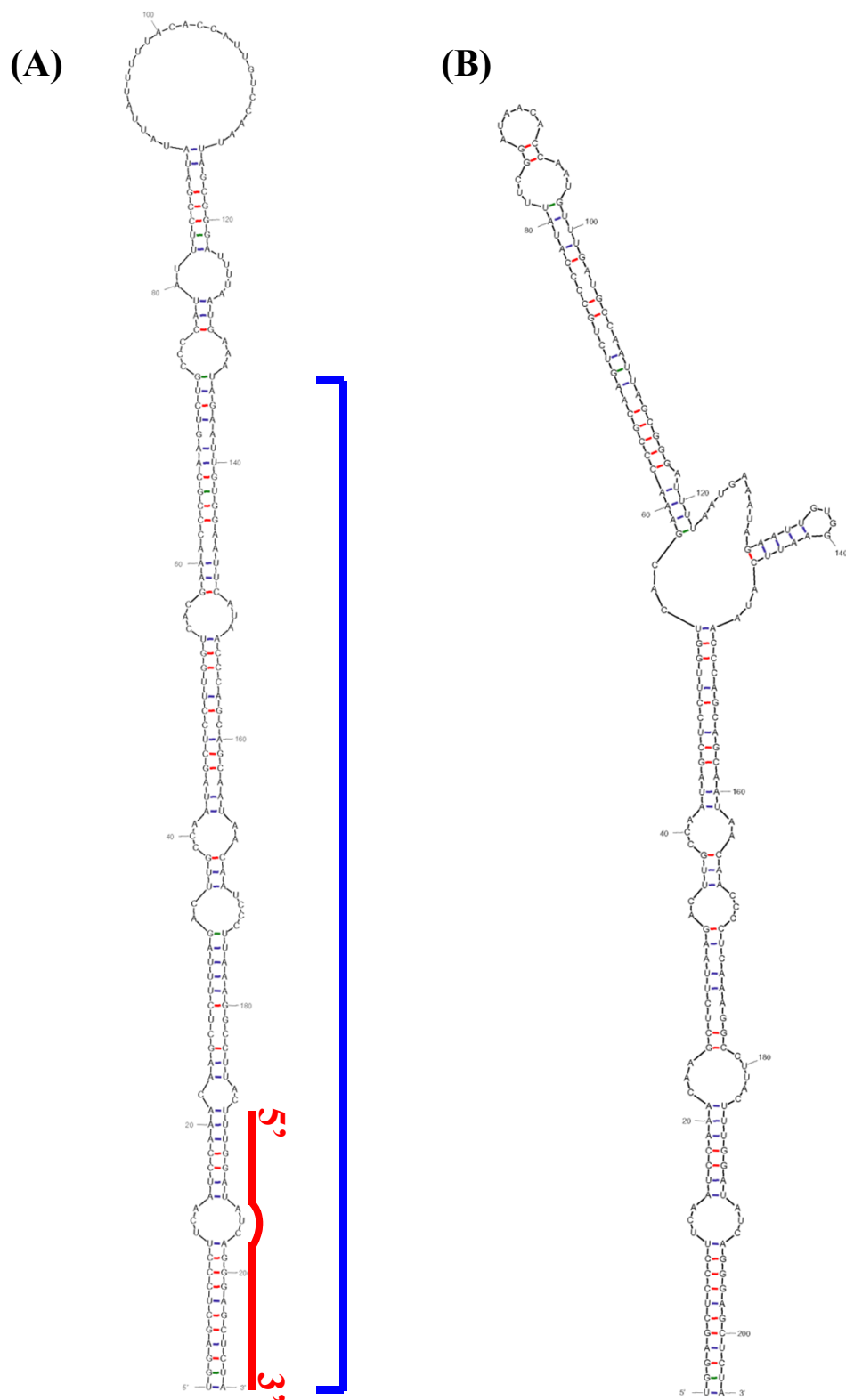

*Phalaenopsis minus*

*Phalaenopsis sumatrana*-type-2

Supplement: Figure S3 — The hairpin secondary structure of the novel pre-miR159 from (A) Phalaenopsis minus ; and (B) P. sumatrana -type 2 with a 10 nt deletion within the terminal loop region. The blue line region represents the fold-back arm of secondary structure for all Phalaenopsis species. The red line region represents the mature miR159. (PDF) [file pone.0114493.s003.pdf]
