# Supplementary material for: Evidence of Purifying Selection and Co-Evolution at the Fold-Back Arm of the Novel Precursor MicroRNA159 Gene in Phalaenopsis Species (Orchidaceae)
Source: PLoS One. 2014 Dec 3;9(12):e114493. doi: 10.1371/journal.pone.0114493 (PMC4254996; doi:10.1371/journal.pone.0114493)
Supplement: Figure S2 — A close-up view of the secondary structure of the novel pre-miR159 from the 42 Phalaenopsis species. (PDF) [file pone.0114493.s002.pdf]

Fig. S2

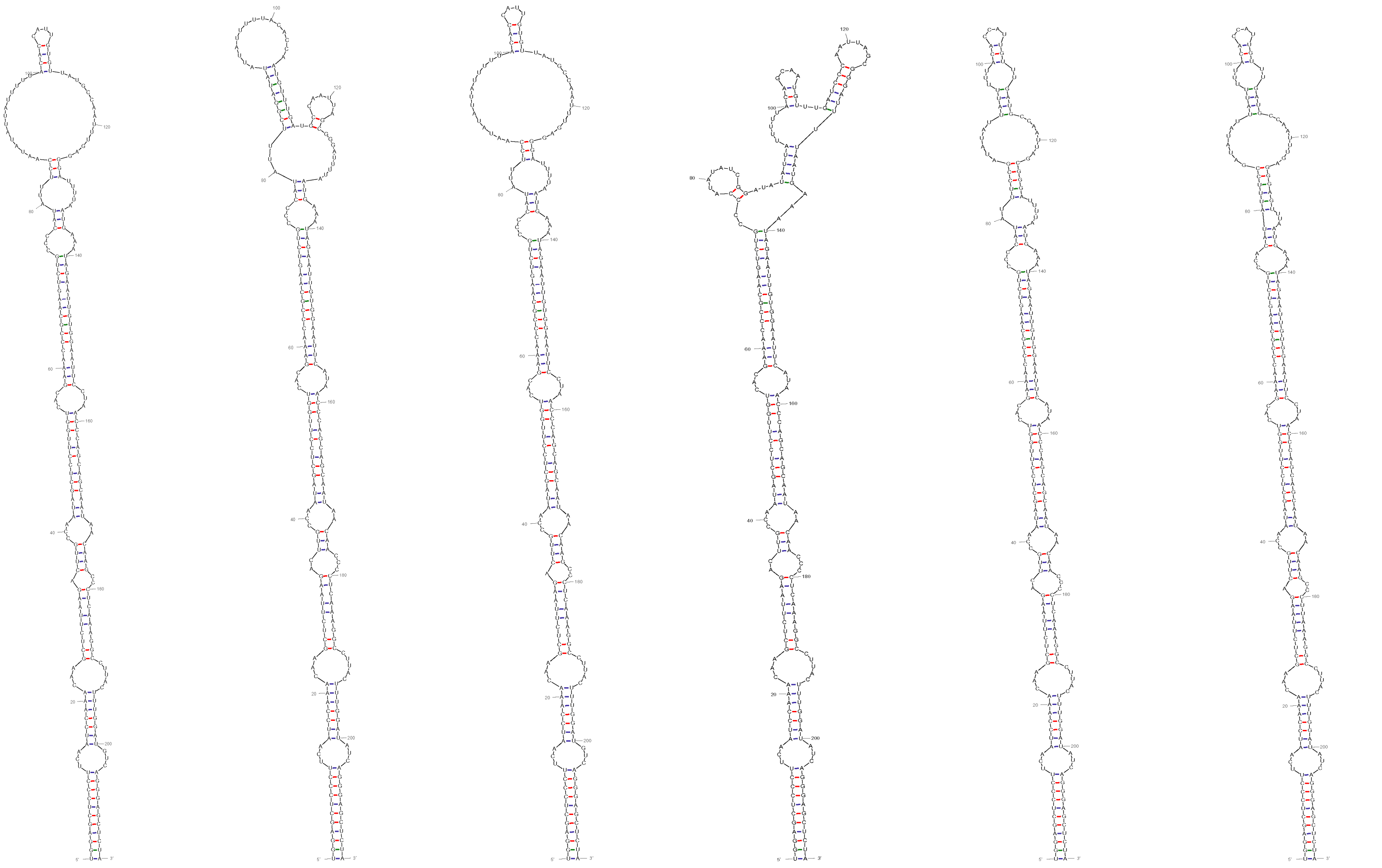

P. amabilis *P. amboinensis* *P. aphrodite* *P. bastianii* *P. braceana* *P. celebensis*

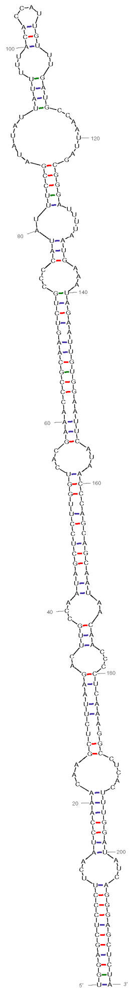

P. chibae

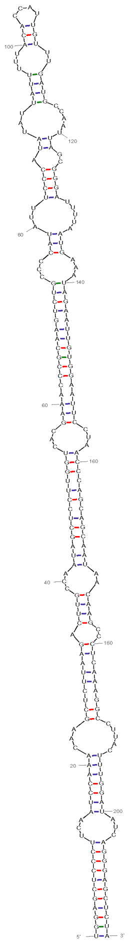

P. cochlearis

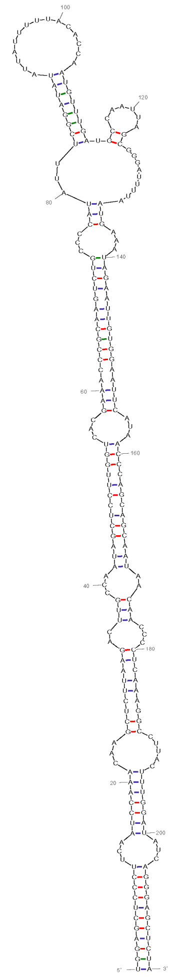

P. corningiana

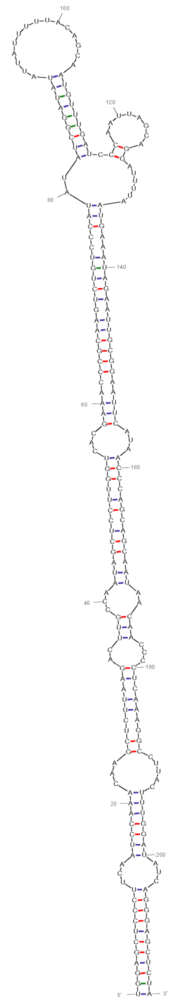

P. cornu-cervi

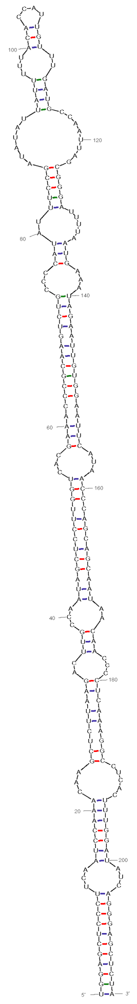

P. deliciosa

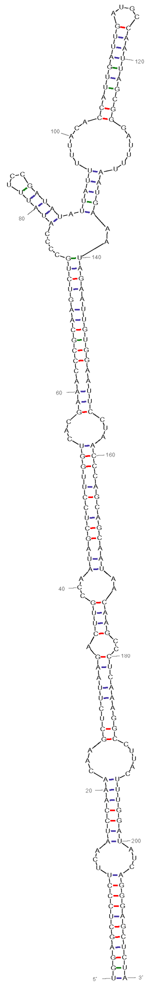

P. doweryensis

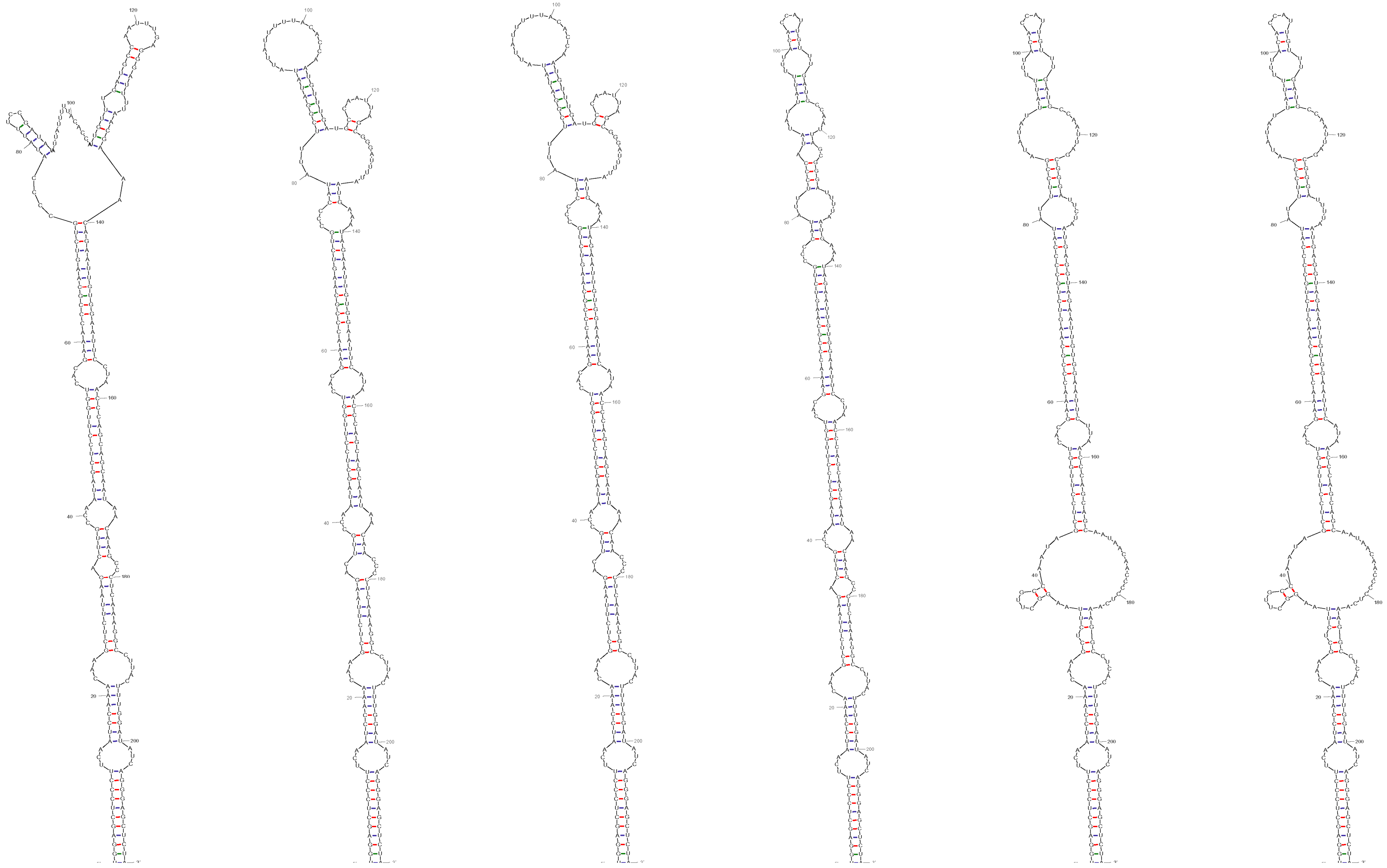

P. equestris *P. fimbriata* *P. floresensis* *P. fuscata* *P. gibbosa*-type-1 *P. gibbosa*-type-2

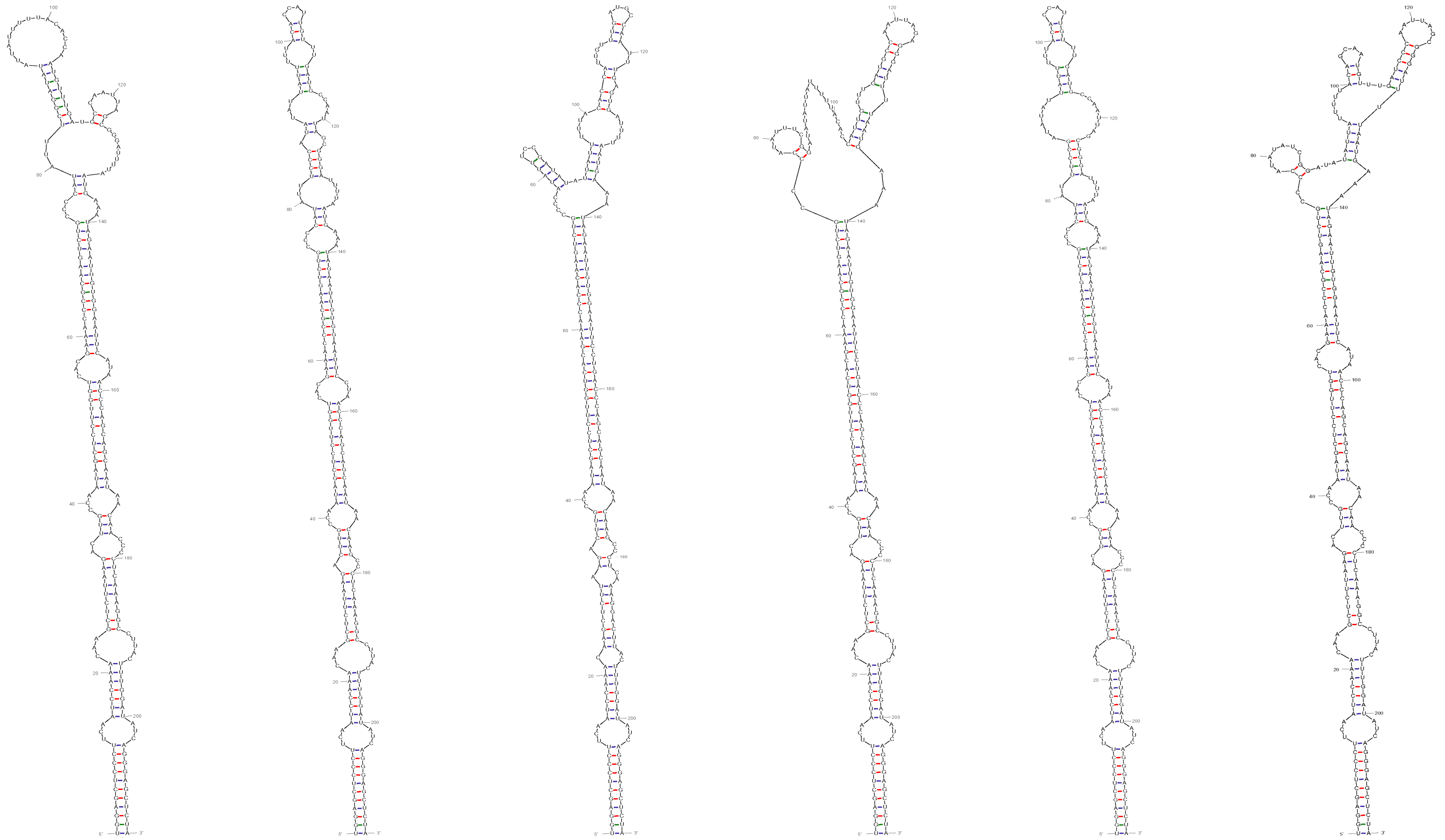

P. inscriptiosinensis *P. kunstleri* *P. lindenii*-type-1 *P. lindenii*-type-2 *P. lowii* *P. lueddemanniana*

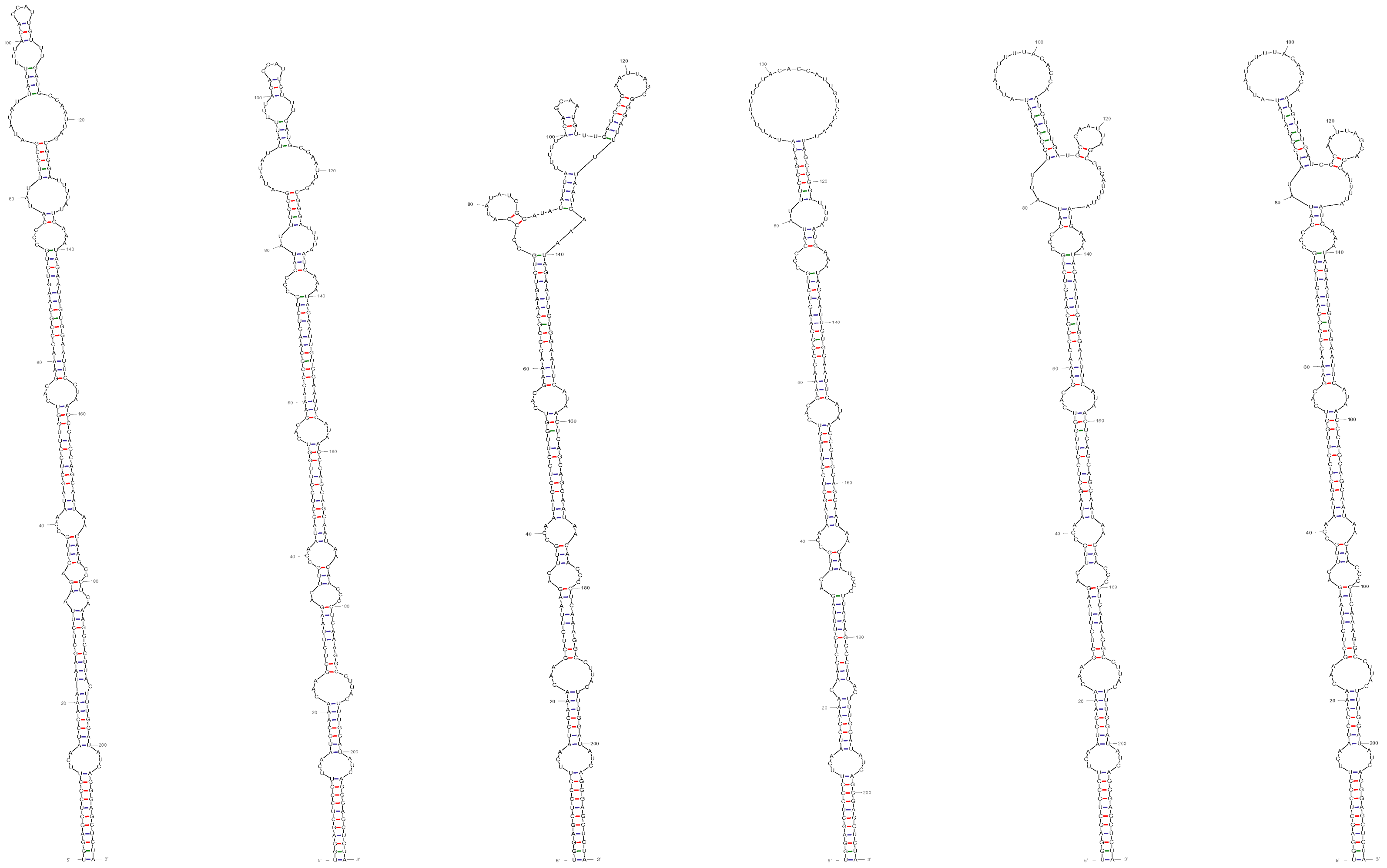

P. maculata *P. mannii* *P. micholitzii* *P. minus* *P. modesta* *P. pantherina*

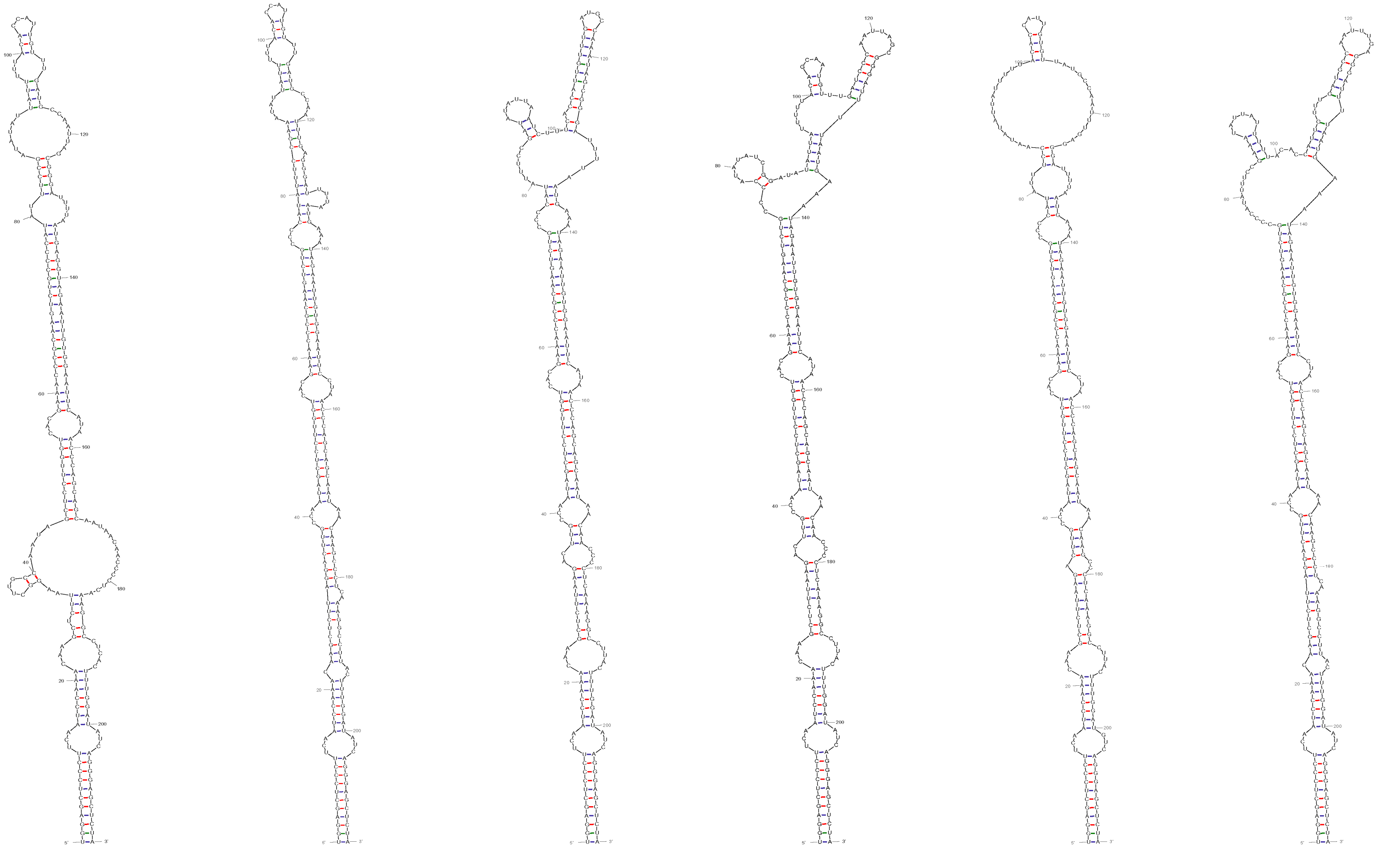

P. parishii *P. philippinensis* *P. pulcherrima* *P. pultra* *P. sanderiana* *P. schilleriana*

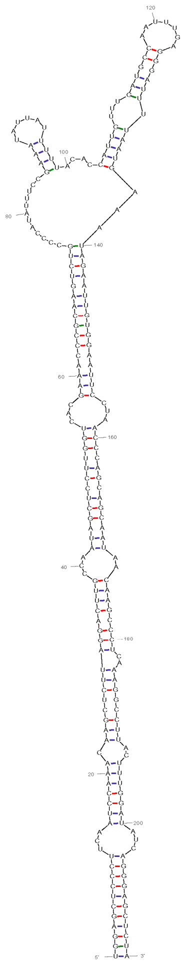

P. stuartiana

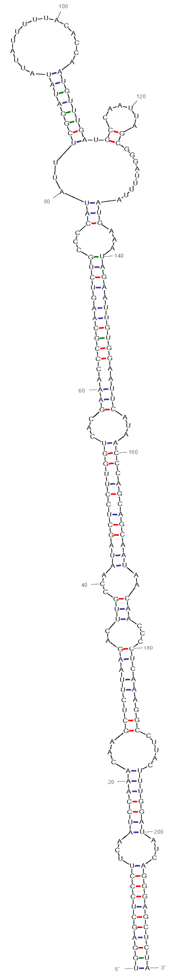

P. sumatrana-type-1

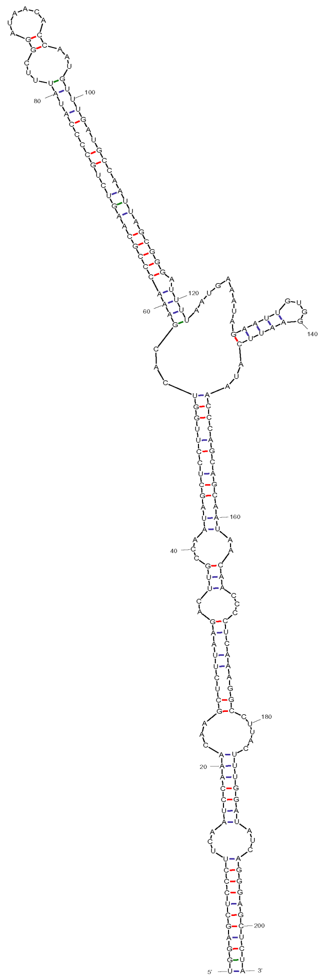

P. sumatrana-type-2

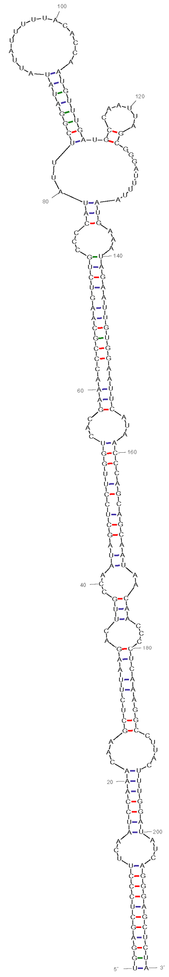

P. tetraspis

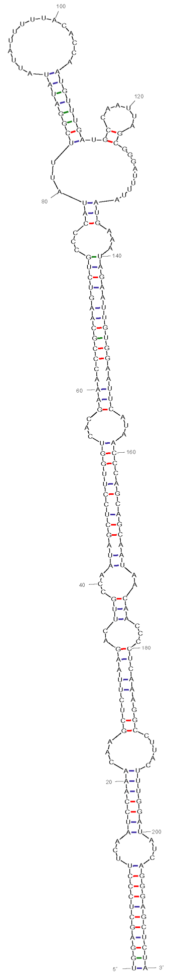

P. venosa

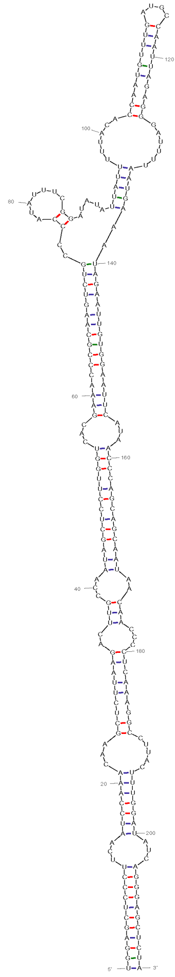

P. violacea

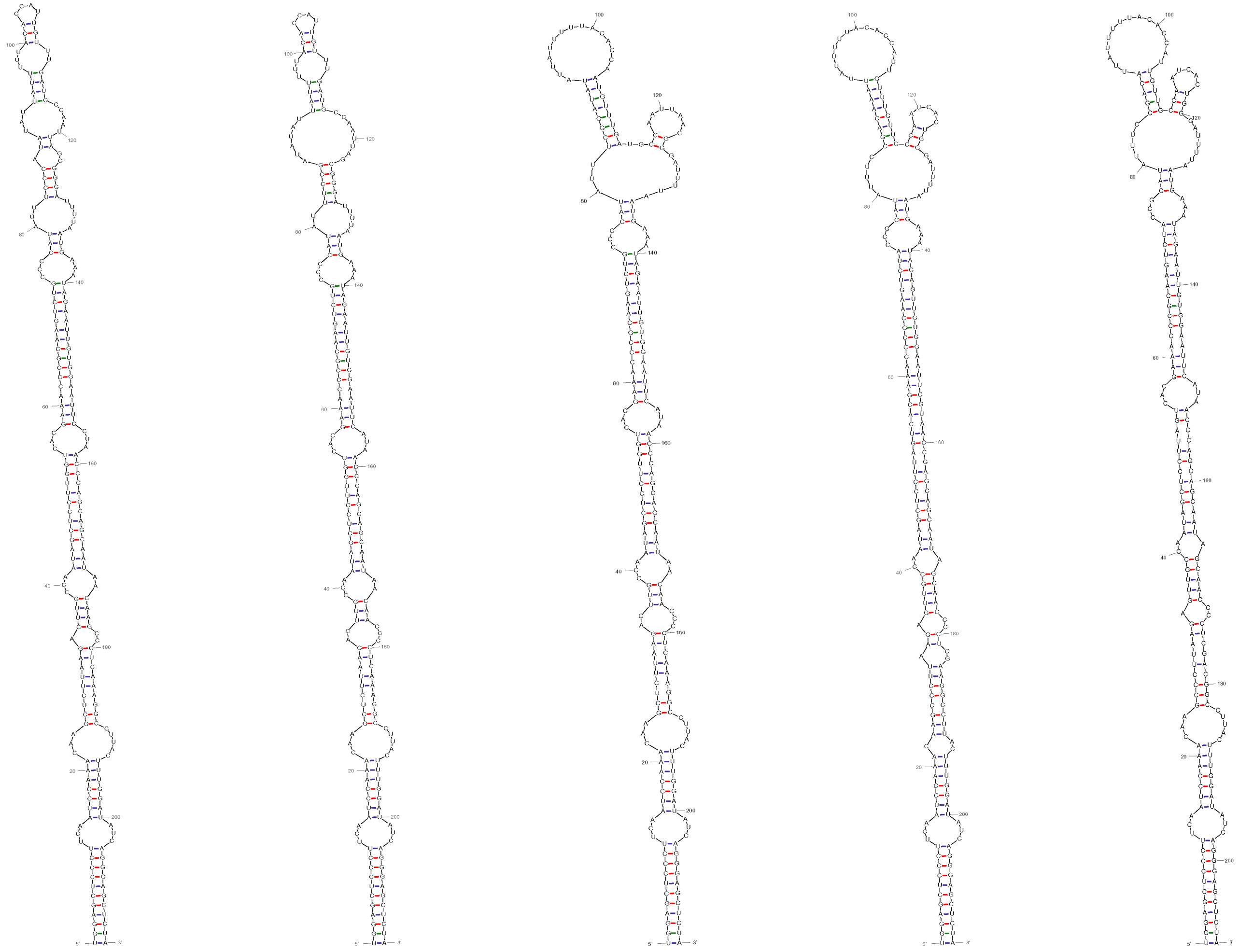

P. viridis *P. wilsonii* *P. zebrina* *Para. laycockii* *Para. serpentilingua*
