# Supplementary material for: Evidence of Purifying Selection and Co-Evolution at the Fold-Back Arm of the Novel Precursor MicroRNA159 Gene in Phalaenopsis Species (Orchidaceae)
Source: PLoS One. 2014 Dec 3;9(12):e114493. doi: 10.1371/journal.pone.0114493 (PMC4254996; doi:10.1371/journal.pone.0114493)
Supplement: Table S2 — The maximum-likelihood relative rate test in the 5' Fold-back arm of the novel pre-miR159 gene of 42 Phalaenopsis species. Pairwise comparisons of Nucleotide substitution rate (above diagonal) and P-value (below diagonal) between species deduced from the 5' Fold-back arm of the novel pre-miR159 gene. (PDF) [file pone.0114493.s005.pdf]

Supplementary Table S2.The maximum-likelihood relative rate test in 5' Fold-back arm of the pre-miR159a gene (Nucleotide substitution rate (above the diagonal) and P-value (below the diagonal))

	P._amabilis	P._amboinensis	P._aphrodite	P._bastianii	P._braceana	P._celebensis	P._chibae	P._cochleari	P._corningiana	P._cornu-cervi	P._deliciosa	P._doweryensis	P._equestris	P._fimbriata	P._floresensis	P._fuscata
P._amabilis		6.14	6.21	6.16	6.15	6.15	6.16	6.16	6.15	6.16	6.15	6.15	6.15	6.15	6.15	6.15
P._amboinensis	0.993		6.15	6.15	6.16	6.16	6.15	6.16	6.16	6.16	6.16	6.16	6.16	6.15	6.15	6.15
P._aphrodite	1.000	0.989		6.15	6.15	6.15	6.15	6.15	6.16	6.16	6.15	6.15	6.15	6.15	6.16	6.16
P._bastianii	0.993	0.989	0.989		6.15	6.16	6.15	6.15	6.16	6.15	6.16	6.16	6.15	6.15	6.16	6.15
P._braceana	0.989	0.989	0.989	0.989		6.15	6.15	6.16	6.15	6.15	6.16	6.15	6.16	6.15	6.15	6.16
P._celebensis	0.989	0.990	0.989	0.989	0.989		6.15	6.16	6.15	6.16	6.16	6.15	6.16	6.15	6.16	6.16
P._chibae	0.989	0.989	0.989	0.989	0.989	0.989		6.15	6.15	6.15	6.16	6.15	6.15	6.15	6.16	6.16
P._cochlearis	0.990	0.989	0.989	0.989	0.989	0.989	0.989		6.16	6.15	6.15	6.15	6.15	6.15	6.15	6.15
P._corningiana	0.989	0.990	0.989	0.989	0.989	0.989	0.989	0.990		6.16	6.15	6.15	6.15	6.16	6.15	6.15
P._cornu-cervi	0.989	0.990	0.990	0.989	0.989	0.989	0.989	0.989	0.989		6.15	6.16	6.16	6.15	6.16	6.15
P._deliciosa	0.989	0.990	0.989	0.989	0.989	0.990	0.989	0.989	0.989	0.989		6.15	6.15	6.15	6.15	6.16
P._doweryensis	0.989	0.990	0.989	0.990	0.989	0.989	0.989	0.989	0.989	0.989	0.989		6.15	6.15	6.15	6.15
P._equestris	0.989	0.990	0.989	0.989	0.989	0.989	0.989	0.989	0.989	0.990	0.989	0.989		6.15	6.15	6.15
P._fimbriata	0.989	0.989	0.989	0.989	0.989	0.989	0.989	0.989	0.989	0.989	0.989	0.989	0.989		6.15	6.15
P._floresensis	0.989	0.989	0.989	0.989	0.989	0.989	0.989	0.989	0.989	0.989	0.989	0.989	0.989	0.989		6.15
P._fuscata	0.989	0.989	0.990	0.989	0.989	0.990	0.990	0.989	0.989	0.989	0.989	0.989	0.989	0.989	0.989	
P._gibbosa-type-1	0.255	0.255	0.255	0.255	0.255	0.255	0.255	0.255	0.255	0.255	0.255	0.255	0.255	0.255	0.255	0.255
P._gibbosa-type-2	0.255	0.255	0.255	0.255	0.255	0.255	0.255	0.255	0.255	0.255	0.255	0.255	0.255	0.255	0.255	0.255
P._inscriptiosinensis	1.000	1.000	1.000	1.000	1.000	1.000	1.000	1.000	1.000	1.000	1.000	1.000	1.000	1.000	1.000	1.000
P._kunstleri	0.989	0.990	0.989	0.989	0.989	0.989	0.989	0.989	0.989	0.989	0.990	0.989	0.989	0.989	0.989	0.989
P._lindenii-type-1	0.255	0.255	0.255	0.255	0.255	0.255	0.255	0.255	0.255	0.255	0.255	0.255	0.255	0.255	0.255	0.255
P._lindenii-type-2	1.000	1.000	1.000	1.000	1.000	1.000	1.000	1.000	1.000	1.000	1.000	1.000	1.000	1.000	1.000	1.000
P._lowii	0.989	0.989	0.989	0.989	0.989	0.990	0.989	0.990	0.989	0.989	0.989	0.989	0.989	0.989	0.989	0.989
P._lueddemanniana	0.989	0.989	0.989	0.989	0.989	0.989	0.989	0.990	0.989	0.989	0.989	0.989	0.989	0.989	0.989	0.989
P._maculata	0.255	0.255	0.255	0.255	0.255	0.255	0.255	0.255	0.255	0.255	0.255	0.255	0.255	0.255	0.255	0.255
P._mannii	1.000	1.000	1.000	1.000	1.000	1.000	1.000	1.000	1.000	1.000	1.000	1.000	1.000	1.000	1.000	1.000
P._micholitzii	0.989	0.990	0.990	0.989	0.990	0.990	0.990	0.989	0.989	0.989	0.989	0.989	0.990	0.990	0.990	0.990
P._minus	0.242	0.242	0.242	0.242	0.242	0.242	0.242	0.242	0.242	0.242	0.242	0.242	0.242	0.242	0.242	0.242
P._modesta	1.000	1.000	1.000	1.000	1.000	1.000	1.000	1.000	1.000	1.000	1.000	1.000	1.000	1.000	1.000	1.000
P._pantherina	0.991	0.991	0.992	0.992	0.992	0.991	0.991	0.991	0.992	0.991	0.992	0.992	0.991	0.991	0.991	0.992
P._parishii	0.255	0.255	0.255	0.255	0.255	0.255	0.255	0.255	0.255	0.255	0.255	0.255	0.255	0.255	0.255	0.255
P._philippinensis	0.255	0.255	0.255	0.255	0.255	0.255	0.255	0.255	0.255	0.255	0.255	0.255	0.255	0.255	0.255	0.255
P._pulcherrima	1.000	1.000	1.000	1.000	1.000	1.000	1.000	1.000	1.000	1.000	1.000	1.000	1.000	1.000	1.000	1.000
P._pulchra	0.989	0.990	0.989	0.990	0.989	0.989	0.989	0.989	0.989	0.989	0.989	0.989	0.989	0.990	0.989	0.989
P._sanderiana	0.989	0.990	0.989	0.990	0.990	0.990	0.989	0.989	0.989	0.989	0.989	0.989	0.989	0.989	0.989	0.989
P._schilleriana	0.255	0.255	0.255	0.255	0.255	0.255	0.255	0.255	0.255	0.255	0.255	0.255	0.255	0.255	0.255	0.255
P._stuartiana	0.255	0.255	0.255	0.255	0.255	0.255	0.255	0.255	0.255	0.255	0.255	0.255	0.255	0.255	0.255	0.255
P._sumatrana-type-1	1.000	1.000	1.000	1.000	1.000	1.000	1.000	1.000	1.000	1.000	1.000	1.000	1.000	1.000	1.000	1.000
P._sumatrana-type-2	0.989	0.989	0.990	0.989	0.989	0.989	0.989	0.990	0.989	0.989	0.989	0.989	0.989	0.989	0.989	0.989
P._tetraspis	0.989	0.989	0.990	0.989	0.989	0.989	0.990	0.990	0.990	0.989	0.989	0.989	0.989	0.989	0.989	0.989
P._venosa	0.989	0.989	0.990	0.989	0.989	0.989	0.990	0.989	0.989	0.990	0.989	0.989	0.989	0.990	0.989	0.990
P._violacea	0.989	0.989	0.989	0.989	0.990	0.989	0.989	0.989	0.989	0.990	0.989	0.989	0.989	0.989	0.989	0.990
P._viridis	0.990	0.989	0.989	0.989	0.990	0.989	0.989	0.989	0.989	0.990	0.990	0.989	0.989	0.989	0.989	0.989
P._wilsonii	0.989	0.990	0.990	0.989	0.989	0.989	0.989	0.990	0.989	0.989	0.989	0.989	0.990	0.989	0.990	0.989
P._zebrina	0.989	0.989	0.990	0.989	0.989	0.989	0.990	0.989	0.989	0.989	0.989	0.989	0.989	0.989	0.990	0.989

	P._gibbosa-type-1	P._gibbosa-type-	P._inscriptiosinensis	P._kunstleri	P._lindenii-type-1	P._lindenii-type-2	P._lowii	P._lueddemanniana	P._maculata	P._mannii	P._micholitzii	P._minus	P._modesta	P._pantherin	P._parishii
P._amabilis	8.22	8.24	6.33	6.15	8.22	6.32	6.15	6.16	8.23	6.35	6.15	3.04	6.17	6.16	8.23
P._amboinensis	8.23	8.24	6.32	6.16	8.23	6.35	6.16	6.15	8.22	6.32	6.16	3.04	6.17	6.16	8.22
P._aphrodite	8.22	8.24	6.32	6.16	8.22	6.32	6.15	6.15	8.22	6.32	6.16	3.04	6.17	6.16	8.22
P._bastianii	8.22	8.24	6.33	6.16	8.23	6.33	6.15	6.16	8.22	6.31	6.15	3.04	6.17	6.16	8.23
P._braceana	8.23	8.25	6.33	6.15	8.23	6.33	6.15	6.15	8.23	6.33	6.16	3.04	6.17	6.16	8.23
P._celebensis	8.23	8.25	6.33	6.16	8.22	6.31	6.16	6.15	8.22	6.32	6.16	3.04	6.17	6.16	8.22
P._chibae	8.23	8.25	6.32	6.16	8.23	6.35	6.15	6.16	8.22	6.31	6.16	3.04	6.17	6.16	8.23
P._cochlearis	8.22	8.24	6.33	6.15	8.22	6.32	6.16	6.16	8.22	6.31	6.15	3.04	6.17	6.16	8.22
P._corningiana	8.23	8.24	6.32	6.15	8.23	6.33	6.15	6.15	8.22	6.32	6.15	3.04	6.17	6.16	8.23
P._cornu-cervi	8.22	8.24	6.33	6.15	8.23	6.33	6.15	6.16	8.22	6.31	6.15	3.04	6.17	6.16	8.22
P._deliciosa	8.22	8.24	6.33	6.16	8.22	6.31	6.15	6.15	8.23	6.33	6.15	3.04	6.17	6.16	8.22
P._doweryensis	8.22	8.24	6.33	6.15	8.22	6.32	6.15	6.16	8.23	6.33	6.16	3.04	6.17	6.16	8.23
P._equestris	8.23	8.25	6.32	6.15	8.23	6.33	6.15	6.16	8.22	6.31	6.16	3.04	6.17	6.16	8.23
P._fimbriata	8.22	8.24	6.33	6.16	8.23	6.33	6.16	6.15	8.23	6.33	6.16	3.04	6.17	6.16	8.23
P._floresensis	8.22	8.24	6.33	6.15	8.22	6.32	6.15	6.16	8.22	6.31	6.16	3.04	6.17	6.16	8.22
P._fuscata	8.22	8.24	6.33	6.16	8.22	6.31	6.15	6.15	8.23	6.33	6.16	3.04	6.17	6.16	8.23
P._gibbosa-type-1		8.36	8.28	8.19	10.65	8.50	8.31	8.26	10.52	8.49	8.31	4.12	8.26	8.19	8.47
P._gibbosa-type-2	1.000		8.19	8.26	10.53	8.49	8.31	8.20	10.64	8.51	8.32	4.13	8.26	8.19	8.47
P._inscriptiosinensis	0.255	0.255		6.26	8.22	6.32	6.16	6.15	8.23	6.33	6.16	3.04	6.17	6.16	8.22
P._kunstleri	0.255	0.255	1.000		8.23	6.33	6.15	6.16	8.23	6.33	6.15	3.04	6.17	6.16	8.23
P._lindenii-type-1	1.000	1.000	0.255	0.255		8.25	8.28	8.19	10.66	8.50	8.31	4.12	8.26	8.19	10.66
P._lindenii-type-2	0.255	0.255	1.000	1.000	0.255		6.32	6.16	8.23	6.35	6.16	3.04	6.17	6.16	8.22
P._lowii	0.255	0.255	0.990	0.989	0.255	1.000		6.16	8.23	6.35	6.15	3.04	6.17	6.16	8.23
P._lueddemanniana	0.255	0.255	0.989	0.989	0.255	0.989	0.989		8.22	6.31	6.16	3.04	6.17	6.16	8.22
P._maculata	1.000	1.000	0.255	0.255	1.000	0.255	0.255	0.255		8.25	8.28	4.13	8.26	8.19	10.66
P._mannii	0.255	0.255	1.000	1.000	0.255	1.000	1.000	1.000	0.255		6.32	3.05	6.17	6.16	8.23
P._micholitzii	0.255	0.255	0.989	0.989	0.255	0.989	0.989	0.990	0.255	1.000		3.04	6.17	6.16	8.23
P._minus	0.962	0.962	0.242	0.242	0.962	0.242	0.242	0.242	0.962	0.242	0.242		3.05	3.05	4.11
P._modesta	0.255	0.255	1.000	1.000	0.255	1.000	1.000	1.000	0.255	1.000	1.000	0.242		6.17	8.22
P._pantherina	0.255	0.255	0.991	0.992	0.255	0.992	0.992	0.991	0.255	0.992	0.991	0.242	1.000		8.23
P._parishii	1.000	1.000	0.255	0.255	1.000	0.255	0.255	0.255	1.000	0.255	0.255	0.963	0.255	0.255	
P._philippinensis	1.000	1.000	0.255	0.255	1.000	0.255	0.255	0.255	1.000	0.255	0.255	0.962	0.255	0.255	1.000
P._pulcherrima	0.255	0.255	1.000	1.000	0.255	1.000	1.000	1.000	0.255	1.000	1.000	0.242	1.000	1.000	0.255
P._pulchra	0.255	0.255	0.989	0.989	0.255	0.990	0.989	0.989	0.255	0.989	0.989	0.242	0.989	0.989	0.255
P._sanderiana	0.255	0.255	0.990	0.989	0.255	0.989	0.989	0.989	0.255	0.989	0.989	0.242	0.989	0.990	0.255
P._schilleriana	1.000	1.000	0.255	0.255	1.000	0.255	0.255	0.255	1.000	0.255	0.255	0.963	0.255	0.255	1.000
P._stuartiana	1.000	1.000	0.255	0.255	1.000	0.255	0.255	0.255	1.000	0.255	0.255	0.962	0.255	0.255	1.000
P._sumatrana-type-1	0.255	0.255	1.000	1.000	0.255	1.000	1.000	1.000	0.255	1.000	1.000	0.242	1.000	1.000	0.255
P._sumatrana-type-2	0.255	0.255	0.989	0.989	0.255	0.990	0.989	0.989	0.255	0.990	0.989	0.242	0.989	0.989	0.255
P._tetraspis	0.255	0.255	0.989	0.989	0.255	0.989	0.989	0.990	0.255	0.990	0.989	0.242	0.990	0.989	0.255
P._venosa	0.255	0.255	0.989	0.989	0.255	0.989	0.989	0.990	0.255	0.989	0.989	0.242	0.990	0.989	0.255
P._violacea	0.255	0.255	0.989	0.989	0.255	0.989	0.990	0.990	0.255	0.989	0.989	0.242	0.990	0.989	0.255
P._viridis	0.255	0.255	0.989	0.989	0.255	0.989	0.989	0.989	0.255	0.989	0.990	0.242	0.990	0.990	0.255
P._wilsonii	0.255	0.255	0.990	0.989	0.255	0.989	0.989	0.989	0.255	0.989	0.989	0.242	0.990	0.989	0.255
P._zebrina	0.255	0.255	0.990	0.989	0.255	0.989	0.989	0.989	0.255	0.989	0.989	0.242	0.989	0.989	0.255

	P._philippinensis	P._pulcherrima	P._pulchra	P._sanderiana	P._schilleriana	P._stuartiana	P._sumatrana-type-	P._sumatrana-type	P._tetraspis	P._venosa	P._violacea	P._viridis	P._wilsonii	P._zebrina
P._amabilis	8.25	6.33	6.15	6.16	8.23	8.25	6.33	6.15	6.15	6.15	6.16	6.16	6.15	6.15
P._amboinensis	8.24	6.33	6.16	6.16	8.23	8.25	6.33	6.15	6.15	6.15	6.15	6.16	6.16	6.15
P._aphrodite	8.24	6.33	6.15	6.16	8.23	8.25	6.32	6.16	6.16	6.16	6.15	6.16	6.16	6.16
P._bastianii	8.25	6.33	6.16	6.16	8.22	8.24	6.32	6.16	6.15	6.15	6.15	6.15	6.15	6.15
P._braceana	8.25	6.33	6.16	6.16	8.22	8.24	6.32	6.15	6.15	6.16	6.16	6.16	6.15	6.15
P._celebensis	8.24	6.32	6.16	6.16	8.23	8.25	6.33	6.15	6.15	6.15	6.15	6.15	6.16	6.15
P._chibae	8.25	6.33	6.15	6.15	8.23	8.25	6.33	6.16	6.16	6.16	6.15	6.15	6.16	6.16
P._cochlearis	8.24	6.33	6.15	6.16	8.23	8.25	6.32	6.16	6.16	6.15	6.15	6.16	6.16	6.15
P._corningiana	8.25	6.33	6.15	6.15	8.23	8.25	6.33	6.16	6.16	6.15	6.15	6.15	6.16	6.15
P._cornu-cervi	8.24	6.32	6.15	6.16	8.23	8.25	6.33	6.15	6.16	6.16	6.16	6.16	6.15	6.16
P._deliciosa	8.24	6.33	6.15	6.16	8.23	8.25	6.33	6.16	6.15	6.15	6.16	6.16	6.15	6.15
P._doweryensis	8.25	6.32	6.15	6.15	8.22	8.24	6.33	6.15	6.16	6.15	6.16	6.15	6.15	6.16
P._equestris	8.25	6.33	6.15	6.16	8.23	8.25	6.32	6.15	6.15	6.15	6.15	6.16	6.16	6.15
P._fimbriata	8.25	6.33	6.16	6.15	8.22	8.24	6.34	6.15	6.16	6.16	6.15	6.16	6.15	6.16
P._floresensis	8.24	6.32	6.15	6.16	8.23	8.25	6.33	6.15	6.15	6.15	6.15	6.16	6.16	6.16
P._fuscata	8.25	6.33	6.15	6.15	8.23	8.25	6.33	6.15	6.16	6.16	6.16	6.15	6.15	6.16
P._gibbosa-type-1	10.57	8.49	8.31	8.20	10.65	10.61	8.50	8.31	8.20	8.27	8.19	8.27	8.19	8.26
P._gibbosa-type-2	10.56	8.49	8.31	8.20	10.65	10.61	8.50	8.31	8.28	8.19	8.27	8.19	8.26	8.19
P._inscriptiosinensis	8.24	6.33	6.16	6.16	8.23	8.25	6.33	6.15	6.15	6.16	6.15	6.16	6.16	6.16
P._kunstleri	8.25	6.32	6.15	6.16	8.23	8.25	6.33	6.15	6.15	6.16	6.15	6.15	6.15	6.16
P._lindenii-type-1	10.61	8.50	8.31	8.28	10.60	10.57	8.49	8.31	8.20	8.27	8.19	8.26	8.19	8.26
P._lindenii-type-2	8.24	6.33	6.16	6.15	8.23	8.25	6.33	6.16	6.15	6.16	6.15	6.15	6.15	6.15
P._lowii	8.25	6.33	6.15	6.15	8.23	8.25	6.32	6.15	6.15	6.16	6.16	6.15	6.15	6.16
P._lueddemanniana	8.24	6.33	6.16	6.15	8.22	8.24	6.33	6.16	6.16	6.16	6.16	6.15	6.15	6.16
P._maculata	10.61	8.50	8.31	8.20	10.65	10.61	8.50	8.31	8.20	8.27	8.19	8.26	8.19	8.26
P._mannii	8.25	6.33	6.16	6.15	8.23	8.25	6.33	6.16	6.16	6.15	6.16	6.15	6.15	6.15
P._micholitzii	8.25	6.33	6.15	6.16	8.23	8.25	6.33	6.15	6.16	6.15	6.16	6.16	6.15	6.16
P._minus	4.12	3.05	3.05	3.05	4.11	4.12	3.05	3.05	3.05	3.05	3.05	3.05	3.05	3.05
P._modesta	8.24	6.34	6.15	6.16	8.23	8.25	6.33	6.16	6.16	6.16	6.16	6.16	6.16	6.15
P._pantherina	8.25	6.33	6.16	6.16	8.23	8.25	6.33	6.15	6.15	6.15	6.16	6.16	6.15	6.15
P._parishii	10.59	8.49	8.31	8.20	10.64	10.61	8.50	8.31	8.20	8.27	8.19	8.27	8.19	8.26
P._philippinensis		8.19	8.26	8.19	8.47	8.36	8.28	8.19	8.26	8.19	8.26	8.19	8.26	8.19
P._pulcherrima	0.255		6.26	6.15	8.23	8.25	6.32	6.16	6.16	6.16	6.16	6.15	6.15	6.16
P._pulchra	0.255	1.000		6.15	8.23	8.25	6.33	6.15	6.15	6.15	6.16	6.16	6.15	6.15
P._sanderiana	0.255	0.989	0.989		8.22	8.24	6.33	6.16	6.16	6.16	6.16	6.16	6.16	6.16
P._schilleriana	1.000	0.255	0.255	0.255		8.36	8.28	8.19	8.26	8.19	8.26	8.19	8.26	8.19
P._stuartiana	1.000	0.255	0.255	0.255	1.000		8.26	8.19	8.26	8.19	8.26	8.19	8.26	8.19
P._sumatrana-type-1	0.255	1.000	1.000	1.000	0.255	0.255		6.26	6.15	6.16	6.15	6.16	6.16	6.16
P._sumatrana-type-2	0.255	0.989	0.989	0.990	0.255	0.255	1.000		6.16	6.16	6.15	6.15	6.15	6.16
P._tetraspis	0.255	0.990	0.989	0.990	0.255	0.255	0.989	0.990		6.15	6.16	6.15	6.15	6.16
P._venosa	0.255	0.990	0.989	0.990	0.255	0.255	0.989	0.990	0.989		6.16	6.15	6.16	6.16
P._violacea	0.255	0.990	0.989	0.990	0.255	0.255	0.989	0.989	0.989	0.990		6.16	6.15	6.15
P._viridis	0.255	0.989	0.990	0.990	0.255	0.255	0.989	0.989	0.989	0.989	0.990		6.15	6.16
P._wilsonii	0.255	0.989	0.989	0.990	0.255	0.255	0.990	0.989	0.989	0.989	0.989	0.989		6.16
P._zebrina	0.255	0.989	0.989	0.990	0.255	0.255	0.990	0.989	0.989	0.990	0.989	0.989	0.990	
