# Supplementary material for: Evidence of Purifying Selection and Co-Evolution at the Fold-Back Arm of the Novel Precursor MicroRNA159 Gene in Phalaenopsis Species (Orchidaceae)
Source: PLoS One. 2014 Dec 3;9(12):e114493. doi: 10.1371/journal.pone.0114493 (PMC4254996; doi:10.1371/journal.pone.0114493)
Supplement: Table S4 — The maximum-likelihood relative rate test in the 3' Fold-back arm of the novel pre-miR159 gene of 42 Phalaenopsis species. Pairwise comparisons of nucleotide substitution rate (above diagonal) and P-value (below diagonal) between species deduced from the 3' Fold-back arm of the novel pre-miR159 gene. (PDF) [file pone.0114493.s007.pdf]

Supplementary Table S4.The maximum-likelihood relative rate test in 3' Fold-back arm of the pre-miR159a gene (Nucleotide substitution rate (above the diagonal) and P-value (below the diagonal))

	P._amabilis	*P._amboinensis*	*P._aphrodite*	*P._bastianii*	*P._braceana*	*P._celebensis*	*P._chibae*	*P._cochlearis*	*P._corningiana*	*P._cornu-cervi*	*P._deliciosa*	*P._doweryensis*	*P._equestris*	*P._fimbriata*	*P._floresensis*
P._amabilis		3.09	2.80	3.12	3.12	3.57	3.86	2.84	3.09	3.86	3.86	2.84	3.57	3.08	3.09
P._amboinensis	0.077		3.08	8.46	8.64	3.09	10.66	2.30	8.32	10.35	10.36	2.30	3.08	8.46	8.64
P._aphrodite	1.000	0.077		3.09	3.09	3.57	3.86	2.84	3.09	3.86	3.86	2.84	3.57	3.09	3.09
P._bastianii	0.077	1.000	0.077		8.46	3.09	10.66	2.30	8.32	10.35	10.37	2.30	3.08	8.46	8.64
P._braceana	0.077	0.991	0.077	1.000		3.09	10.66	2.30	8.32	10.34	10.35	2.30	3.08	8.46	8.64
P._celebensis	1.000	0.077	1.000	0.077	0.077		3.86	2.85	3.09	3.86	3.86	2.84	3.57	3.09	3.09
P._chibae	0.417	0.260	0.417	0.260	0.260	0.417		3.09	10.48	12.81	10.73	3.09	3.86	10.55	10.55
P._cochlearis	0.256	0.179	0.256	0.179	0.179	0.256	0.755		2.30	3.09	3.09	2.06	2.80	2.30	2.30
P._corningiana	0.077	0.988	0.077	0.988	0.988	0.077	0.260	0.179		10.37	10.37	2.30	3.08	8.46	8.64
P._cornu-cervi	0.417	0.260	0.417	0.260	0.260	0.417	0.998	0.755	0.260		12.34	3.09	3.86	10.58	10.51
P._deliciosa	0.417	0.260	0.417	0.260	0.260	0.417	1.000	0.755	0.260	1.000		3.09	3.86	10.55	10.55
P._doweryensis	0.256	0.179	0.256	0.179	0.179	0.256	0.755	1.000	0.179	0.755	0.755		2.81	2.30	2.30
P._equestris	1.000	0.077	1.000	0.077	0.077	1.000	0.417	0.256	0.077	0.417	0.417	0.256		3.09	3.09
P._fimbriata	0.077	1.000	0.077	1.000	1.000	0.077	0.260	0.179	1.000	0.260	0.260	0.179	0.077		8.46
P._floresensis	0.077	0.991	0.077	0.991	0.991	0.077	0.260	0.179	0.991	0.260	0.260	0.179	0.077	1.000	
P._fuscata	0.256	0.179	0.256	0.179	0.179	0.256	0.755	1.000	0.179	0.755	0.755	1.000	0.256	0.179	0.179
P._gibbosa-type-1	0.539	0.157	0.539	0.157	0.157	0.539	0.350	0.960	0.157	0.712	0.350	0.960	0.539	0.157	0.157
P._gibbosa-type-2	0.417	0.260	0.417	0.260	0.260	0.417	0.994	0.755	0.260	1.000	0.995	0.755	0.417	0.260	0.260
P._inscriptiosinensis	0.077	1.000	0.077	1.000	1.000	0.077	0.260	0.179	1.000	0.260	0.260	0.179	0.077	0.999	1.000
P._kunstleri	0.256	0.179	0.256	0.179	0.179	0.256	0.755	1.000	0.179	0.755	0.755	1.000	0.256	0.179	0.179
P._lindenii-type-1	0.538	**0.039**	0.538	**0.039**	**0.039**	0.538	0.240	0.103	**0.039**	0.240	0.240	0.103	0.538	**0.039**	**0.039**
P._lindenii-type-2	0.536	0.157	0.536	0.157	0.157	0.536	0.712	0.967	0.157	0.712	0.712	0.967	0.536	0.157	0.157
P._lowii	0.077	0.993	0.077	0.993	0.993	0.077	0.260	0.179	0.993	0.260	0.260	0.179	0.077	0.993	0.993
P._lueddemanniana	0.077	0.995	0.077	0.994	0.996	0.077	0.260	0.179	0.997	0.260	0.260	0.179	0.077	0.997	0.995
P._maculata	0.256	0.179	0.256	0.179	0.179	0.256	0.755	1.000	0.179	0.755	0.755	1.000	0.256	0.179	0.179
P._mannii	0.077	0.988	0.077	0.988	0.988	0.077	0.260	0.179	0.988	0.260	0.260	0.179	0.077	0.988	0.988
P._micholitzii	0.417	0.260	0.417	0.260	0.260	0.417	0.998	0.754	0.260	1.000	0.999	0.754	0.417	0.260	0.260
P._minus	0.569	0.111	0.569	0.112	0.112	0.242	0.560	0.992	0.112	0.559	0.559	0.992	0.569	0.111	0.111
P._modesta	0.417	0.260	0.417	0.260	0.260	0.417	0.999	0.755	0.260	1.000	1.000	0.755	0.417	0.260	0.260
P._pantherina	0.077	1.000	0.077	1.000	1.000	0.077	0.260	0.179	1.000	0.260	0.260	0.179	0.077	1.000	1.000
P._parishii	0.417	0.260	0.417	0.260	0.260	0.417	0.996	0.755	0.260	0.999	0.996	0.755	0.417	0.260	0.260
P._philippinensis	0.256	0.179	0.256	0.179	0.179	0.256	0.755	1.000	0.179	0.755	0.755	1.000	0.256	0.179	0.179
P._pulcherrima	0.077	0.988	0.077	0.988	0.988	0.077	0.260	0.179	0.988	0.260	0.260	0.179	0.077	0.988	0.988
P._pulchra	0.077	1.000	0.077	1.000	1.000	0.077	0.260	0.179	1.000	0.260	0.260	0.179	0.077	1.000	1.000
P._sanderiana	1.000	0.077	0.999	0.077	0.077	1.000	0.417	0.256	0.077	0.417	0.417	0.256	1.000	0.077	0.077
P._schilleriana	0.256	0.179	0.256	0.179	0.179	0.256	0.755	1.000	0.179	0.755	0.755	1.000	0.256	0.179	0.179
P._stuartiana	0.256	0.179	0.256	0.179	0.179	0.256	0.755	1.000	0.179	0.755	0.755	1.000	0.256	0.179	0.179
P._sumatrana-type-1	0.077	0.988	0.077	0.988	0.988	0.077	0.260	0.179	0.988	0.260	0.260	0.179	0.077	0.988	0.988
P._sumatrana-type-2	0.077	1.000	0.077	1.000	1.000	0.077	0.260	0.179	1.000	0.260	0.260	0.179	0.077	1.000	1.000
P._tetraspis	0.077	1.000	0.077	1.000	1.000	0.077	0.260	0.179	1.000	0.260	0.260	0.179	0.077	1.000	1.000
P._venosa	0.077	1.000	0.077	1.000	1.000	0.077	0.260	0.179	1.000	0.260	0.260	0.179	0.077	1.000	1.000
P._violacea	0.077	1.000	0.077	1.000	1.000	0.077	0.260	0.179	1.000	0.260	0.260	0.179	0.077	1.000	1.000
P._viridis	0.256	0.179	0.256	0.179	0.179	0.256	0.755	1.000	0.179	0.755	0.755	1.000	0.256	0.179	0.179
P._wilsonii	0.077	0.988	0.077	0.988	0.988	0.077	0.260	0.179	0.988	0.260	0.260	0.179	0.077	0.988	0.988
P._zebrina	0.077	1.000	0.077	1.000	1.000	0.077	0.260	0.179	1.000	0.260	0.260	0.179	0.077	1.000	1.000

	P._fuscata	*P._gibbosa*-type-1	*P._gibbosa*-type-2	*P._inscriptiosinensis*	*P._kunstleri*	*P._lindenii*-type-1	*P._lindenii*-type-2	*P._lowii*	*P._lueddemanniana*	*P._maculata*	*P._mannii*	*P._micholitzii*	*P._minus*	*P._modesta*	*P._pantherina*
P._amabilis	2.85	4.12	3.86	3.08	2.85	2.63	3.50	3.09	3.09	2.85	3.09	3.86	4.27	3.86	3.09
P._amboinensis	2.30	4.68	10.36	8.47	2.30	**2.29**	4.68	8.38	8.64	2.30	8.32	10.35	12.43	10.59	8.58
P._aphrodite	2.85	4.12	3.86	3.09	2.85	2.63	3.50	3.09	3.09	2.85	3.09	3.86	4.27	3.86	3.08
P._bastianii	2.30	4.68	10.27	8.46	2.30	**2.29**	4.68	8.38	8.64	2.30	8.32	10.34	12.45	10.61	8.58
P._braceana	2.30	4.68	10.34	8.47	2.30	**2.29**	4.68	8.38	8.64	2.30	8.32	10.35	12.45	10.61	8.58
P._celebensis	2.85	4.12	3.86	3.09	2.85	2.63	3.50	3.10	3.09	2.85	3.09	3.86	3.36	3.86	3.09
P._chibae	3.09	4.61	10.64	10.37	3.09	2.87	5.90	10.54	10.54	3.09	10.51	12.85	14.99	12.87	10.76
P._cochlearis	2.06	3.37	3.09	2.30	2.06	2.06	2.75	2.30	2.30	2.06	2.30	3.14	3.43	3.09	2.30
P._corningiana	2.30	4.68	10.36	8.47	2.30	**2.29**	4.68	8.38	8.64	2.30	8.32	10.34	12.47	10.62	8.58
P._cornu-cervi	3.09	5.90	12.76	10.79	3.09	2.87	5.90	10.54	10.55	3.09	10.50	12.88	14.84	12.89	10.75
P._deliciosa	3.09	4.66	10.66	10.52	3.09	2.87	5.90	10.54	10.54	3.09	10.52	12.87	14.84	12.89	10.75
P._doweryensis	2.06	3.37	3.09	2.30	2.06	2.06	2.75	2.30	2.30	2.06	2.30	3.14	3.43	3.09	2.30
P._equestris	2.85	4.12	3.86	3.09	2.85	2.63	3.50	3.08	3.12	2.85	3.09	3.86	4.27	3.86	3.09
P._fimbriata	2.30	4.68	10.35	8.44	2.30	**2.29**	4.68	8.38	8.64	2.30	8.32	10.35	12.43	10.59	8.58
P._floresensis	2.30	4.68	10.36	8.47	2.30	**2.29**	4.68	8.38	8.64	2.30	8.32	10.35	12.43	10.59	8.58
P._fuscata		3.39	3.09	2.30	2.06	2.06	2.76	2.30	2.30	2.06	2.30	3.09	3.43	3.09	2.30
P._gibbosa-type-1	0.960		4.66	4.68	3.38	3.05	6.30	4.74	4.68	3.38	4.68	5.95	6.93	5.93	4.73
P._gibbosa-type-2	0.755	0.350		10.53	3.09	2.87	5.90	10.54	10.55	3.09	10.56	12.83	14.99	12.88	10.75
P._inscriptiosinensis	0.179	0.157	0.260		2.30	**2.29**	4.68	8.38	8.64	2.30	8.32	10.34	12.43	10.59	8.58
P._kunstleri	1.000	0.960	0.755	0.179		2.06	2.75	2.30	2.30	2.06	2.30	3.14	3.43	3.09	2.30
P._lindenii-type-1	0.103	0.289	0.240	**0.039**	0.103		2.00	**2.29**	**2.29**	2.07	**2.30**	2.87	3.19	2.87	**2.29**
P._lindenii-type-2	0.967	1.000	0.712	0.157	0.967	0.100		4.68	4.68	2.76	4.68	5.95	6.91	5.93	4.73
P._lowii	0.179	0.157	0.260	0.993	0.179	**0.039**	0.157		8.38	2.30	8.32	10.34	12.42	10.59	8.58
P._lueddemanniana	0.179	0.157	0.260	0.995	0.179	**0.039**	0.157	0.993		2.30	8.32	10.34	12.42	10.59	8.58
P._maculata	1.000	0.960	0.755	0.179	1.000	0.103	0.967	0.179	0.179		2.30	3.14	3.43	3.09	2.30
P._mannii	0.179	0.157	0.260	0.988	0.179	**0.039**	0.157	0.988	0.988	0.179		10.66	12.40	10.59	8.58
P._micholitzii	0.755	0.712	0.998	0.260	0.754	0.240	0.712	0.260	0.260	0.754	0.260		15.15	10.82	10.56
P._minus	0.993	0.847	0.560	0.111	0.992	0.353	0.846	0.111	0.111	0.992	0.111	0.560		15.09	12.59
P._modesta	0.755	0.712	0.999	0.260	0.755	0.240	0.712	0.260	0.260	0.755	0.260	1.000	0.560		10.72
P._pantherina	0.179	0.157	0.260	1.000	0.179	**0.039**	0.157	1.000	1.000	0.179	1.000	0.260	0.112	0.260	
P._parishii	0.755	0.350	0.996	0.260	0.755	0.240	0.712	0.260	0.260	0.755	0.260	0.998	0.560	1.000	0.260
P._philippinensis	1.000	0.960	0.755	0.179	1.000	0.103	0.967	0.179	0.179	1.000	0.179	0.755	0.991	0.755	0.179
P._pulcherrima	0.179	0.157	0.260	0.988	0.179	**0.039**	0.157	0.988	0.988	0.179	0.988	0.260	0.112	0.260	0.988
P._pulchra	0.179	0.157	0.260	1.000	0.179	**0.039**	0.157	1.000	1.000	0.179	1.000	0.260	0.112	0.260	1.000
P._sanderiana	0.256	0.539	0.417	0.077	0.256	0.538	0.536	0.077	0.077	0.256	0.077	0.417	0.569	0.417	0.077
P._schilleriana	1.000	0.960	0.755	0.179	1.000	0.103	0.967	0.179	0.179	1.000	0.179	0.755	0.990	0.755	0.179
P._stuartiana	1.000	0.960	0.755	0.179	1.000	0.103	0.968	0.179	0.179	1.000	0.179	0.755	0.991	0.755	0.179
P._sumatrana-type-1	0.179	0.157	0.260	0.988	0.179	**0.039**	0.157	0.988	0.988	0.179	0.988	0.260	0.112	0.260	0.988
P._sumatrana-type-2	0.179	0.157	0.260	1.000	0.179	**0.039**	0.157	1.000	1.000	0.179	1.000	0.260	0.112	0.260	1.000
P._tetraspis	0.179	0.157	0.260	1.000	0.179	**0.039**	0.157	1.000	1.000	0.179	1.000	0.260	0.112	0.260	1.000
P._venosa	0.179	0.157	0.260	1.000	0.179	**0.039**	0.157	1.000	1.000	0.179	1.000	0.260	0.112	0.260	1.000
P._violacea	0.179	0.157	0.260	1.000	0.179	**0.039**	0.157	1.000	1.000	0.179	1.000	0.260	0.112	0.260	1.000
P._viridis	1.000	0.960	0.755	0.179	1.000	0.103	0.967	0.179	0.179	1.000	0.179	0.755	0.990	0.755	0.179
P._wilsonii	0.179	0.157	0.260	0.988	0.179	**0.039**	0.157	0.988	0.988	0.179	0.988	0.260	0.112	0.260	0.988
P._zebrina	0.179	0.157	0.260	1.000	0.179	**0.039**	0.157	1.000	1.000	0.179	1.000	0.260	0.112	0.260	1.000

	P._parishii	*P._philippinensis*	*P._pulcherrima*	*P._pulchra*	*P._sanderiana*	*P._schilleriana*	*P._stuartiana*	*P._sumatrana*-type-1	*P._sumatrana*-type-2	*P._tetraspis*	*P._venosa*	*P._violacea*	*P._viridis*	*P._wilsonii*	*P._zebrina*
P._amabilis	3.86	2.85	3.09	3.09	2.80	2.84	2.84	3.09	3.09	3.09	3.12	3.09	2.81	3.09	3.09
P._amboinensis	10.36	2.30	8.32	8.64	3.09	2.30	2.30	8.32	8.64	8.64	8.64	8.64	2.30	8.32	8.64
P._aphrodite	3.86	2.84	3.09	3.09	2.80	2.84	2.85	3.09	3.09	3.12	3.09	3.12	2.85	3.09	3.09
P._bastianii	10.35	2.30	8.32	8.64	3.09	2.30	2.30	8.32	8.64	8.64	8.64	8.64	2.30	8.32	8.64
P._braceana	10.38	2.30	8.32	8.64	3.09	2.30	2.30	8.32	8.64	8.64	8.64	8.64	2.30	8.32	8.64
P._celebensis	3.86	2.85	3.09	3.12	3.57	2.85	2.81	3.09	3.12	3.09	3.12	3.09	2.85	3.09	3.09
P._chibae	10.66	3.09	10.50	10.55	3.86	3.09	3.09	10.48	10.53	10.54	10.57	10.49	3.09	10.50	10.54
P._cochlearis	3.09	2.06	2.30	2.30	2.81	2.06	2.07	2.30	2.30	2.30	2.30	2.30	2.06	2.30	2.30
P._corningiana	10.38	2.30	8.32	8.64	3.09	2.30	2.30	8.32	8.64	8.64	8.64	8.64	2.30	8.32	8.64
P._cornu-cervi	12.86	3.09	10.55	10.55	3.86	3.09	3.09	10.52	10.56	10.51	10.54	10.55	3.09	10.49	10.55
P._deliciosa	10.66	3.09	10.56	10.50	3.86	3.09	3.09	10.50	10.54	10.55	10.56	10.50	3.09	10.50	10.53
P._doweryensis	3.09	2.06	2.30	2.30	2.81	2.06	2.07	2.30	2.30	2.30	2.30	2.30	2.06	2.30	2.30
P._equestris	3.86	2.84	3.09	3.09	3.57	2.85	2.85	3.09	3.12	3.09	3.09	3.09	2.85	3.09	3.12
P._fimbriata	10.37	2.30	8.32	8.64	3.09	2.30	2.30	8.32	8.64	8.64	8.64	8.64	2.30	8.32	8.64
P._floresensis	10.36	2.30	8.32	8.64	3.09	2.30	2.30	8.32	8.64	8.64	8.64	8.64	2.30	8.32	8.64
P._fuscata	3.09	2.06	2.30	2.30	2.81	2.06	2.07	2.30	2.30	2.30	2.30	2.30	2.06	2.30	2.30
P._gibbosa-type-1	4.66	3.39	4.68	4.68	4.12	3.39	3.39	4.68	4.68	4.68	4.68	4.68	3.38	4.68	4.68
P._gibbosa-type-2	10.67	3.09	10.52	10.53	3.86	3.09	3.09	10.56	10.50	10.54	10.57	10.51	3.09	10.50	10.54
P._inscriptiosinensis	10.37	2.30	8.32	8.64	3.09	2.30	2.30	8.32	8.64	8.64	8.64	8.64	2.30	8.32	8.64
P._kunstleri	3.09	2.06	2.30	2.30	2.81	2.06	2.07	2.30	2.30	2.30	2.30	2.30	2.06	2.30	2.30
P._lindenii-type-1	2.87	2.07	**2.30**	**2.29**	2.64	2.07	2.07	**2.30**	**2.29**	**2.29**	**2.29**	**2.29**	2.07	**2.29**	**2.29**
P._lindenii-type-2	5.94	2.76	4.68	4.68	3.50	2.76	2.76	4.68	4.68	4.68	4.68	4.68	2.76	4.68	4.68
P._lowii	10.36	2.30	8.32	8.64	3.09	2.30	2.30	8.32	8.64	8.64	8.64	8.64	2.30	8.32	8.64
P._lueddemanniana	10.34	2.30	8.32	8.64	3.09	2.30	2.30	8.32	8.64	8.64	8.64	8.64	2.30	8.32	8.64
P._maculata	3.09	2.06	2.30	2.30	2.81	2.06	2.07	2.30	2.30	2.30	2.30	2.30	2.06	2.30	2.30
P._mannii	10.29	2.30	8.32	8.64	3.09	2.30	2.30	8.32	8.64	8.64	8.64	8.64	2.30	8.32	8.64
P._micholitzii	12.84	3.09	10.55	10.56	3.86	3.09	3.09	10.56	10.50	10.53	10.55	10.57	3.09	10.50	10.54
P._minus	15.00	3.45	12.89	12.89	4.27	3.45	3.46	12.89	12.89	12.89	12.89	12.89	3.45	12.89	12.89
P._modesta	12.83	3.09	10.52	10.53	3.86	3.09	3.09	10.50	10.54	10.55	10.55	10.54	3.09	10.50	10.55
P._pantherina	10.38	2.30	8.32	8.64	3.09	2.30	2.30	8.32	8.64	8.64	8.64	8.64	2.30	8.32	8.64
P._parishii		3.09	10.52	10.55	3.86	3.09	3.09	10.48	10.53	10.54	10.55	10.57	3.09	10.52	10.53
P._philippinensis	0.755		2.30	2.30	2.81	2.06	2.07	2.30	2.30	2.30	2.30	2.30	2.06	2.30	2.30
P._pulcherrima	0.260	0.179		8.32	3.08	2.30	2.30	8.32	8.64	8.64	8.64	8.64	2.30	8.32	8.64
P._pulchra	0.260	0.179	0.988		3.09	2.30	2.30	8.32	8.64	8.64	8.64	8.64	2.30	8.32	8.64
P._sanderiana	0.417	0.256	0.077	0.077		2.84	2.85	3.09	3.12	3.09	3.12	3.09	2.85	3.09	3.09
P._schilleriana	0.755	1.000	0.179	0.179	0.256		2.06	2.30	2.30	2.30	2.30	2.30	2.06	2.30	2.30
P._stuartiana	0.755	1.000	0.179	0.179	0.256	1.000		2.30	2.30	2.30	2.30	2.30	2.06	2.30	2.30
P._sumatrana-type-1	0.260	0.179	0.988	0.988	0.077	0.179	0.179		8.32	8.64	8.64	8.64	2.30	8.32	8.64
P._sumatrana-type-2	0.260	0.179	1.000	1.000	0.077	0.179	0.179	0.988		8.64	8.64	8.64	2.30	8.32	8.64
P._tetraspis	0.260	0.179	1.000	1.000	0.077	0.179	0.179	1.000	1.000		8.64	8.64	2.30	8.32	8.64
P._venosa	0.260	0.179	1.000	1.000	0.077	0.179	0.179	1.000	1.000	1.000		8.64	2.30	8.32	8.64
P._violacea	0.260	0.179	1.000	1.000	0.077	0.179	0.179	1.000	1.000	1.000	1.000		2.30	8.32	8.64
P._viridis	0.755	1.000	0.179	0.179	0.256	1.000	1.000	0.179	0.179	0.179	0.179	0.179		2.30	2.30
P._wilsonii	0.260	0.179	0.988	0.988	0.077	0.179	0.179	0.988	0.988	0.988	0.988	0.988	0.179		8.32
P._zebrina	0.260	0.179	1.000	1.000	0.077	0.179	0.179	1.000	1.000	1.000	1.000	1.000	0.179	0.988	
